# Supplementary material for: Edible mycelium bioengineered for enhanced nutritional value and sensory appeal using a modular synthetic biology toolkit
Source: Nat Commun. 2024 Mar 14;15:2099. doi: 10.1038/s41467-024-46314-8 (PMC10940619; doi:10.1038/s41467-024-46314-8)
Supplement: Supplementary file 1 — Supplementary Information [file 41467_2024_46314_MOESM1_ESM.pdf]

Supplementary information

Edible mycelium bioengineered for enhanced nutritional value and sensory appeal using a modular synthetic biology toolkit.

Vayu Maini Rekdal et al.

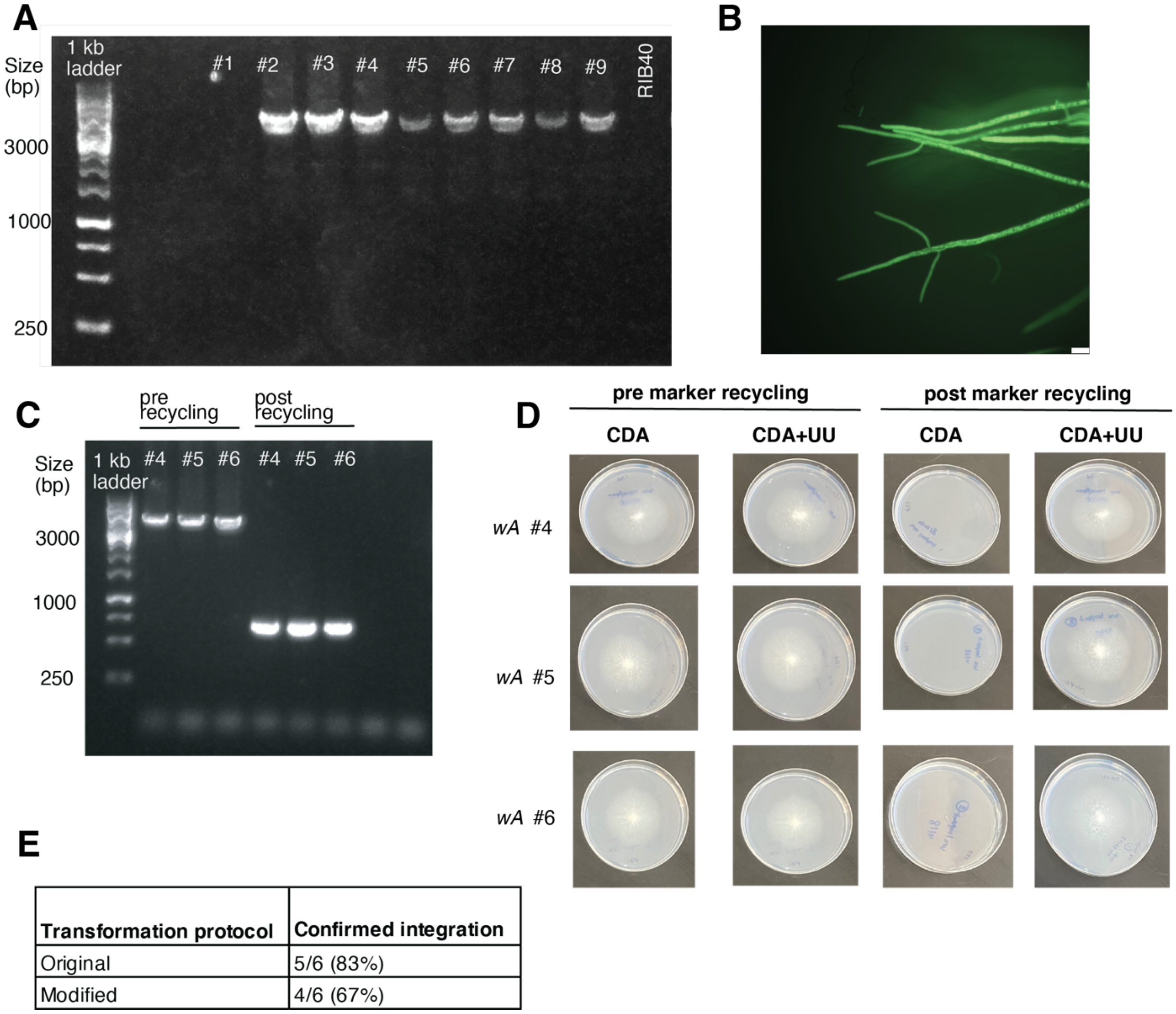

E

Transformation protocol	Confirmed integration
Original	5/6 (83%)
Modified	4/6 (67%)

Supplementary Figure 1. Gene integration and marker excision from the *wA* locus in *A. oryzae* RIB40. A) Following transformation of the RIB40 *pyrG* mutant with RNP complexes and the fixing template harboring a GFP expression cassette under the pAmyB promoter, individual colonies displayed the expected PCR amplicon (~4 kb with primers TamyB-DC-F and wA-in-1R, see Fig. 1C and 1G for details on integration strategy and amplicon size), indicating successful integration at the *wA* locus. B) Transformants displayed the expected expression of GFP from the pAmyB promoter. A representative *wA:gfp* strain is shown. Scale bar = 50 µm. C) Following exposure to 5-FOA, the *pyrG* marker was removed from the *wA* locus. The marker excision was

confirmed for three out of three excised strains and the corresponding parent strains (pre recycling). D) The strains confirmed by PCR also displayed the expected uridine / uracil (UU) auxotrophy. The white colony radiating from the center of the plate indicates growth. E) The protoplast transformation protocol was modified for increased throughout without major losses in efficiency. Compared to the original *A. oryzae* protocol, the modified protocol uses only 50 μL protoplasts, only one addition of PEG, a smaller amount of DNA, and excludes the final heated top agar addition, making the protocol highly compatible with a microplate format for high-throughput manipulations. See materials and methods for details. The integration efficiency was determined by PCR. Six colonies were screened for each protocol.

Supplementary Table 1. Genomic identifiers and associated sgRNAs for *wA*, *niaD*, *yA*, and *pyrG* loci used in RNP-based gene editing in *A. oryzae*. To validate the RNP-based CRISPR-Cas9 method for efficient gene integration and genome modification, we initially targeted loci involved in spore coloration (*wA* and *yA*), nitrate assimilation (*niaD*), and uridine / uracil biosynthesis (*pyrG*). The table displays the gene name, the genomic identifier (from the *A. oryzae* RIB40 genome in the Comprehensive *Aspergillus oryzae* genome database, CAoGD), as well as the sgRNA sequences. The PAM sequence (NGG) is highlighted in red. The same sgRNAs were used for gene editing in *A. oryzae* RIB40 as for the five different *A. oryzae* NRRL strains sequenced in this study (see Supplementary Table 2 for more information about these strains).

**Gene name**	***A. oryzae* RIB40 identifier** (CAoGD)	**5' sgRNA**	**3' sgRNA**
wA	AO090102000545	GCCGCGTGGCCCCTCCATGT TGG	CAAACCGTATATCAGTGCGG TGG
yA	AO090011000755	CCAGCACCAACGCGATCTGG GGG	TCACTAATGGGAAGTATCCA GGG
niaD	AO090012001035	CCTCGTAATTTTACTTTCAT TGG	CCTATCACCCTCGTATGTGC TGG
pyrG	AO090011000868	GACAACCACCAAAGAGCTGC TGG	CTGCAGTATTCTGCCCGGTG AGG

A

Strain	Isolation source	Geographical origin
NRRL1911	Takamine's factory (original strain used for diastase enzyme production)	Japan
NRRL2218	Food, soybean-wheat flour mix	China
NRRL32614	Food factory; koji	Malaysia
NRRL5592	Food, sake koji	Japan
NRRL6574	Hamanatto	Japan

B

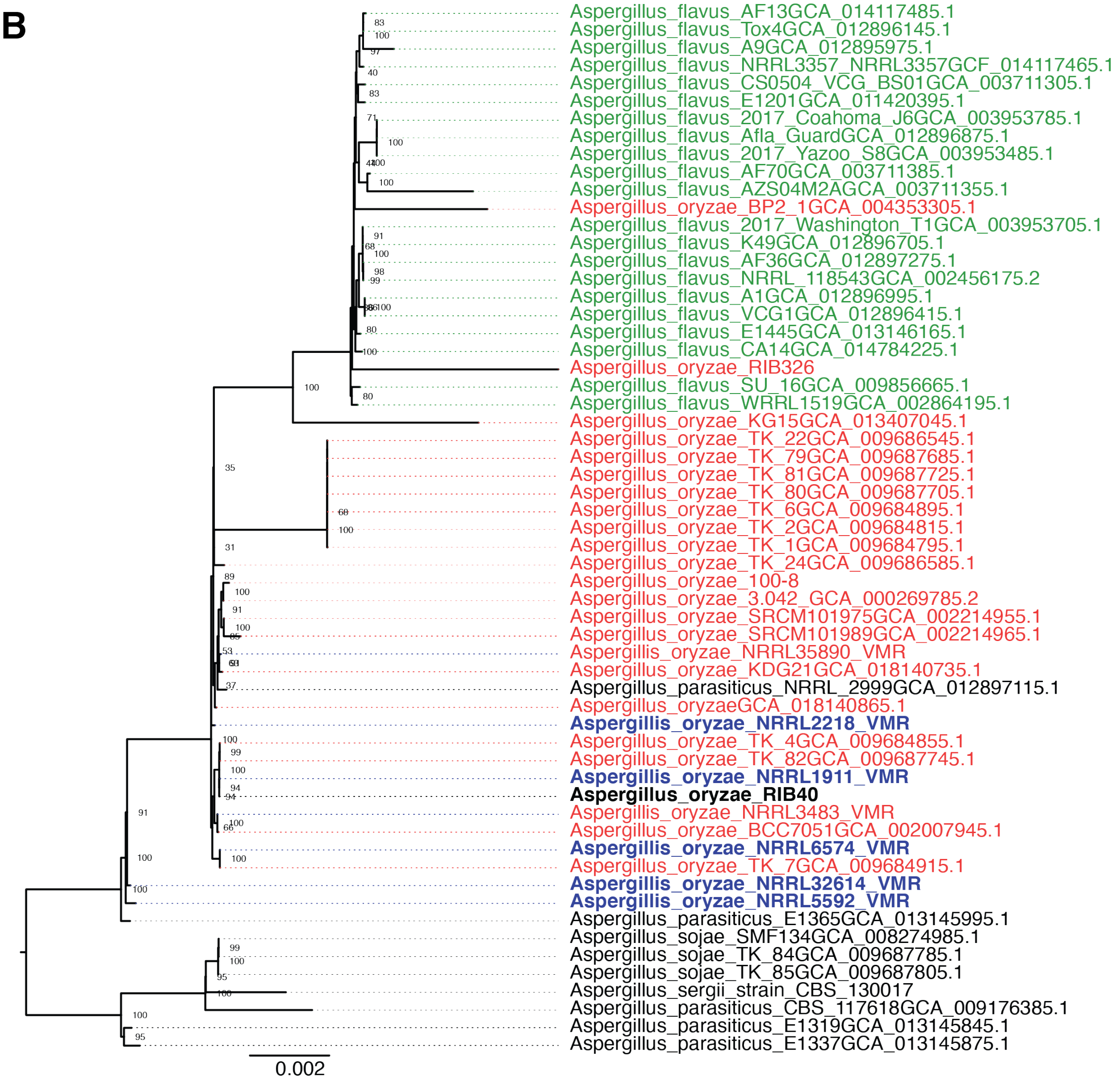

Supplementary Figure 2. Geographical origin, industrial uses, and phylogeny of *A. oryzae* NRRL strains selected for genome modification. A) Geographical information about *A. oryzae* NRRL strains selected for genome modification. The original source information was provided by NRRL and revealed diverse geographical origins and industrial uses. The NRRL 1911 strain was the original producer of the diastase enzyme, for which the first ever patent on a microbial enzyme was granted in the U.S.A. [1]. B) Representative phylogeny of *A. oryzae* NRRL strains. Strains included for comparison are shown in red, while the close relative *Aspergillus flavus* is shown in green. Other *Aspergilli* used for comparison are shown in black. Strains that were whole-genome sequenced and gene edited in this study are highlighted in blue. The genome sequencing information of these strains is described in Supplementary Table 2. The tree was constructed using the protein coding sequences of 167 core genes. See methods for details.

Supplementary Table 2. Genomic information about *A. oryzae* NRRL strains selected for genome modification. The genome sequences of the *A. oryzae* strains obtained from NRRL and sequenced as part of this study have been deposited to the Sequence Read Archive under Bioproject #PRJNA987873. In addition to basic genome metrics, the strains were genomically analyzed for BGCs (biosynthetic gene clusters) to further understand their differences (see methods for details). NRPS = nonribosomal peptide synthase. PKS = polyketide synthase.

	Genome metrics					BGCs			
Strain	Length (bp)	Contigs	G+C content	N50	Coding sequences	Indole	NRPS	PKS	terpene
NRRL 1911	37758747	202	0.47	420259	11554	7	36	30	15
NRR L2218	38464187	197	0.47	666080	11676	7	35	31	15
NRRL 32614	37571356	129	0.48	731545	11546	7	35	31	14
NRRL 5592	38016050	191	0.48	542694	11626	6	35	30	14
NRRL 6574	37496292	193	0.48	424281	11595	7	36	31	13

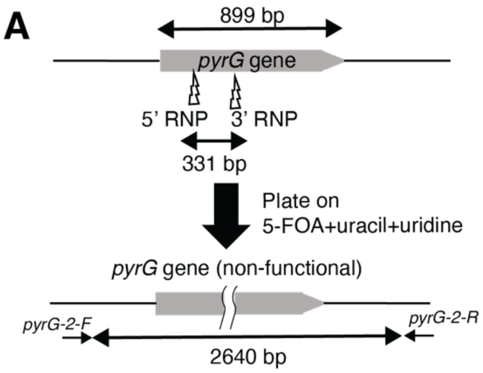

B

Strain	Screened/confirmed auxotrophs
NRRL1911	2/2
NRRL2218	3/3
NRRL32614	2/2
NRRL5592	2/2
NRRL6574	3/3

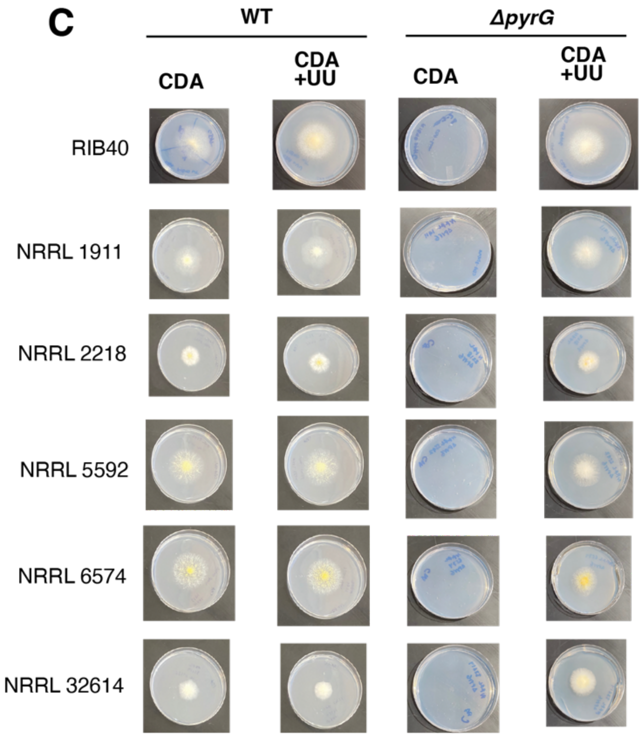

D

Strain	Change in *pyrG* region
NRRL1911	306 bp non-aligned insertion between 5' and 3' RNP cutsites
NRRL2218	308 bp non-aligned insertion between 5' and 3' RNP cutsites
NRRL32614	16 bp non-aligned insertion immediately after 3' RNP cutsite
NRRL6574	Clean 308 bp deletion between 5' and 3' RNP cutsites
NRRL5592	296 bp non-aligned insertion between 5' and 3' RNP cutsites

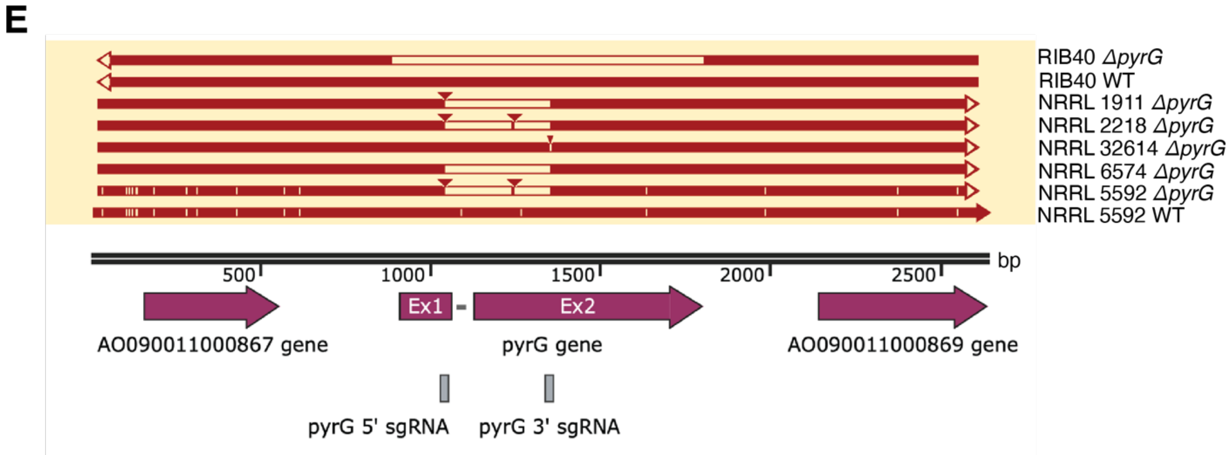

Supplementary Figure 3. Generation of *pyrG* mutants in *A. oryzae* NRRL strains. A) Strategy for *pyrG* mutant generation. Two guide RNAs (sequences are shown in Table S1) were designed to target two different regions of the *pyrG* gene, spaced 399 bp apart. Unlike the transformations for gene integration (shown in Fig. 1C), no DNA template was provided to fix the double-stranded breaks by homology-directed repair. Rather, the endogenous fungal DNA repair machinery had to fix the breaks, potentially causing errors and disrupting the function of the *pyrG* gene. Transformants were plated onto medium supplemented with 5-FOA as well as uridine and uracil, which selects for *pyrG* mutants. B) Two or three transformant colonies for each NRRL strain were screened for the resulting auxotrophy, and all evaluated colonies displayed the phenotype expected for the *pyrG* mutant, suggesting that the mutation and selection method was successful. C) One mutant for each strain was selected for further investigations and subsequent downstream transformations. Whereas the WT strains grew in the presence and absence of supplement, the selected *pyrG* mutants required uracil / uridine (UU) for growth. The colony radiating from the center of the plate indicates growth. Strains were grown for 3-5 days at 30 degrees Celsius. D) The region surrounding *pyrG* was amplified using the primers pyrG-2-F and pyrG-2-R shown in A). The PCR amplicon captured not only the entire coding sequence of *pyrG,* but also the full sequence of the gene 5' to *pyrG* and a portion of the gene located at the 3' end of *pyrG.* Sequence analysis revealed that all *pyrG* mutants carried disruptions between the predicted RNP cut sites. Whereas NRRL 6574 had a clean deletion between the two RNPs, the other strains had insertions of random sequences that could not be aligned to *pyrG*, reflecting erroneous fixing of the DSBs. All these mutations are predicted to render the *pyrG* non-functional. E) Alignment of the PCR amplicons summarized in D). The alignment demonstrates that, as expected, the RIB40 *pyrG* mutant which was provided for this study, has a complete deletion of the entire coding region. For the other

strains, the arrows indicate the site(s) of insertion. Ex1 = exon 1; Ex2 = exon 2 of the *pyrG* gene. There was no off-target mutation observed of the two surrounding genes. The PCR amplicon for the NRRL 5592 wild-type strain is shown because the wild-type NRRL 5592 had a few differences (SNPs) in the amplified region (white bars) that were also present in the *pyrG* mutant, confirming that these observed differences were not a result of CRISPR-Cas9 activity and rather is a unique feature of NRRL 5592 relative to the other strains.

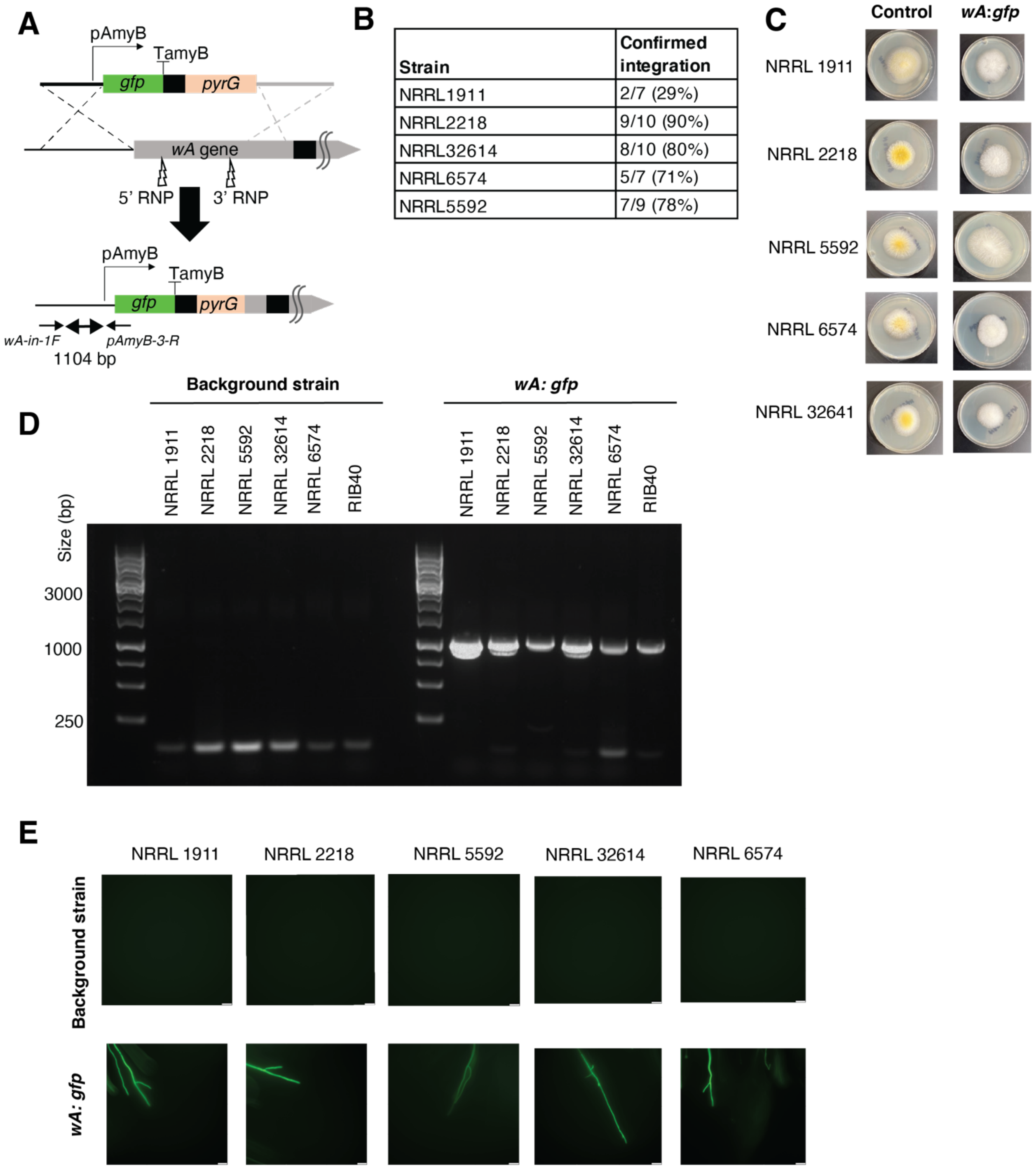

Strain	Confirmed integration
NRRL1911	2/7 (29%)
NRRL2218	9/10 (90%)
NRRL32614	8/10 (80%)
NRRL6574	5/7 (71%)
NRRL5592	7/9 (78%)

Supplementary Figure 4. Gene integration at the *wA* locus in *A. oryzae* NRRL strains. A) Strategy for gene insertion at the *wA* locus in NRRL *pyrG* mutants. The mutants were generated and confirmed according to the strategy described in Supplementary Figure 3, allowing for transformation with a *pyrG*-marker cassette. The targeting strategy was informed by previous

successful gene integration using a plasmid-based CRISPR-Cas9 system[2]. The two squiggly lines indicate that not the whole 6.7-kb gene is shown. Insertion of the GFP cassette was confirmed by primers wA-in-1F (on chromosome) and pAmyB-3-R (on fixing template), yielding a 1104bp amplicon. The same fixing template and sgRNAs as in RIB40 were used in the NRRL strains. B) 7-10 colonies of the transformants were screened for insertion for the NRRL strains. All strains but NRRL 1911 displayed a high efficiency of insertion (>70%) as assessed by PCR. C) Transformants harboring the GFP expression cassette at the *wA* locus also displayed the expected white spore phenotype, which differs from the green-yellow conidia of the wild-type strains. Strains were grown on PDA. D) PCR confirmation of successful insertion at the *wA* locus across all NRRL strains. The RIB40 wild-type and *wA*:*gfp* transformant were included for comparison. All transformants displayed the expected 1104-bp amplicon, which was not detected in the background strains. The ladder is Generuler 1kb ladder (Thermo Scientific). E) NRRL strains harboring the GFP expression cassette at the *wA* locus displayed the expected protein expression, as assessed by fluorescence microscopy. Scale bar = 50 µm.

Supplementary Table 3. Comparison of integration efficiency between one and two sgRNAs across the *wA* and *niaD* loci. A GFP expression cassette was integrated at either the *wA* or *niaD* locus (strategies described in Fig.1 and Supplementary Figure 5), and PCR was used to assess the incorporation of the fixing template at the locus of interest. 10 colonies were randomly selected and evaluated for the diagnostic PCR. Although our method incorporates two RNP complexes based on demonstrated success in other fungi and the goal to maximize the chance of double-stranded breaks, we found no major difference in integration efficiency across the two independent genomic loci, *wA* and *niaD*. Thus, one RNP complex is likely sufficient for successful gene integration and genome editing. The 5' and 3' annotation refers to the sequences shown in Supplementary Table 1.

Locus	RNP complex	Confirmed integration
wA	5'	10/10 (100%)
wA	3'	8/10 (80%)
wA	5' + 3'	9/10 (90%)
niaD	5'	8/10 (80%)
niaD	3'	9/10 (90%)
niaD	5' + 3'	7/10 (70%)

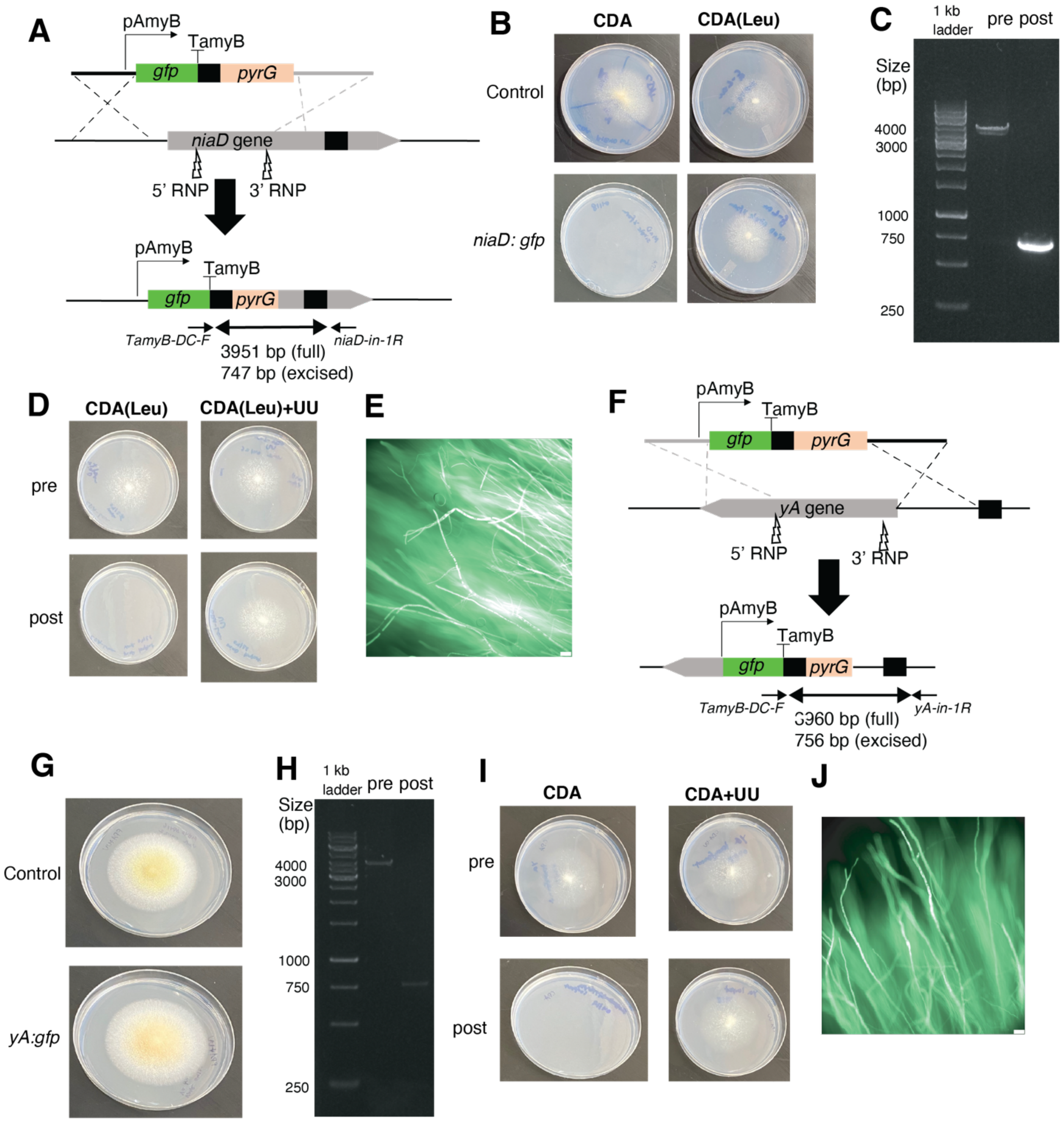

K

Locus	Confirmed integration
niaD	6/6 (100%)
yA	6/6 (100%)

Supplementary Figure 5. Confirmation of successful gene integration and *pyrG* marker excision from the *niaD* and *yA* loci in *A. oryzae* RIB40. A) Strategy for integration of a GFP expression cassette at the *niaD* locus, which is involved in nitrate assimilation in *A. oryzae*. B) The

niaD:gfp strain can use leucine (Leu) as a nitrogen source but is unable to assimilate nitrate, which is the standard nitrogen source in CDA medium. A representative strain is shown. C) The parent and looped-out *niaD:gfp* strain displayed the expected PCR band (primers TamyB-DC-F and niaD-in-1R, described in A), confirming integration at the locus. Ladder is Generuler 1kb ladder (Thermo Scientific). D) The loop-out was accompanied by the expected uracil / uridine auxotrophy, highlighting removal of the *pyrG* gene. The colony radiating from the center of the plate indicates growth. E) The *niaD:gfp* strain also displayed GFP expression. Scale bar = 100 µm. F) Strategy for integration of a GFP expression cassette at the *yA* locus, which is involved in conidial pigmentation in *A. oryzae.* G) The *yA:gfp* strain displays a spore coloration pattern that is more yellow-brown than the yellow-green color of the wild-type RIB40 parent strain. A representative strain is shown. H) The parent and looped-out *yA:gfp* strain displayed the expected PCR band (primers TamyB-DC-F and yA-in-1R, described in F), confirming integration at the locus. Ladder is Generuler 1kb ladder (Thermo Scientific). I) The loop-out of the *pyrG* marker in the *ya:gfp* strain was accompanied by the expected uracil / uridine auxotrophy, highlighting removal of the *pyrG* gene. The colony radiating from the center of the plate indicates growth. J) The *yA:gfp* strain also displayed GFP expression. Scale bar = 100 µm. K) Integration was confirmed in 100% of the tested colonies for each locus, indicating a high editing efficiency similar to that seen at the *wA* locus (Fig. 1 of main text).

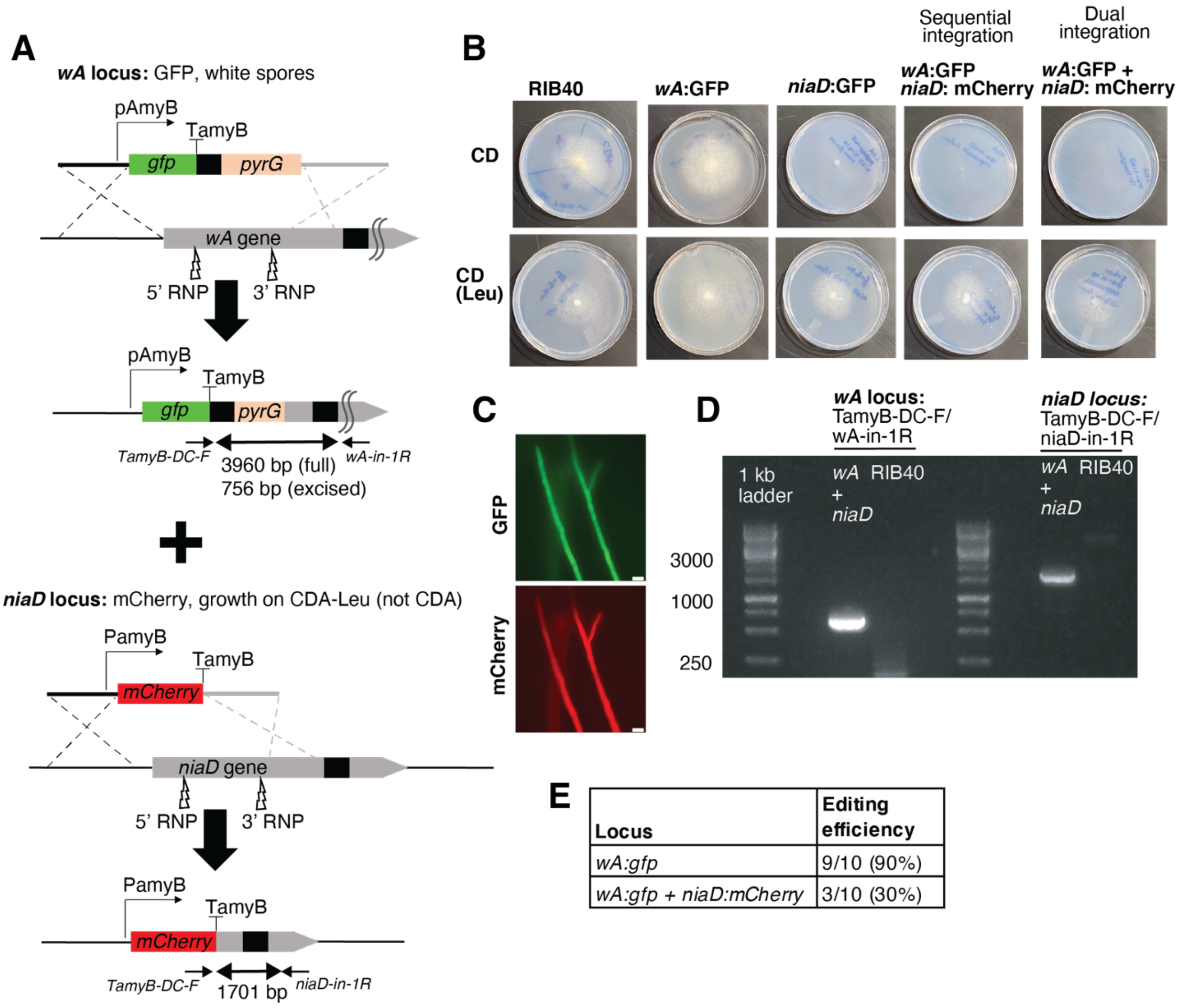

Locus	Editing efficiency
wA:gfp	9/10 (90%)
wA:gfp + niaD:mCherry	3/10 (30%)

Supplementary Figure 6. Simultaneous targeting of *niaD* and *wA* loci in a single transformation. A) Strategy for targeting two loci simultaneously with RNP complexes. The *pyrG* marker was only incorporated in the GFP-containing *wA* fixing template, enabling marker-free editing at the second *niaD* locus, where mCherry was incorporated. B) Confirmation of successful *wA* and *niaD* editing in the simultaneous transformation. A dual integration transformant displayed white spores and the expected nitrate assimilation deficiency phenotype (only growth on CD-Leu). C) *wA:gfp* and *niaD:mCherry* strain expressed both GFP and mCherry in mycelia under the pAmyB promoter. Scale bar = 25 µm. D) A dual integration transformant

was subjected to marker recycling. PCR amplification confirmed the excision of the *pyrG* marker from the *wA* locus (756b), while the *niaD* locus, which did not incorporate a *pyrG* marker in the fixing template, displayed the expected band demonstrating mCherry incorporation (1704bp). As expected, there was no amplification of the background RIB40 strain, as amplification would only occur upon successful integration. The ladder is the Generuler 1kb ladder (Thermo Scientific). E) The efficiency of editing at two simultaneous loci decreased over a single modification at the *wA* locus.

Supplementary Table 4. Candidate neutral loci targeted for evaluation of gene expression. These loci were identified from a larger set computationally identified (in Supplementary data file 1). The *amyA* gene (coding sequence) was included as a control. The PAM sequence (NGG) is highlighted in red. The genomic identifier (beginning with "AO") is from the *A. oryzae* RIB40 genome in the Comprehensive *Aspergillus oryzae* genome database, CAoGD.

**Locus name**	**Chromosome**	**Intergenic region size (bp)**	**5' gene**	**3' gene**	**5' sgRNA sequence**	**3' sgRNA sequence**
chro1_3	1	6503	AO090009000173	AO090009000174	GAAAACAATAGCGAACCCTA CGG	TAAATATAATGCGCTTTGTC CGG
chro1_2	1	5273	AO090005000582	AO090005000581	GTTTCTGTAGCCAACCGCTC CGG	GACGCGTATTGGCCAGGACC TGG
chro2_2	2	4796	AO090003000984	AO090003000985	TGTTGTTAGTCACGGGGGTC GGG	TGTGCTCGTCTCTGGGAACC CGG
chro3_1	3	7294	AO090023000902	AO090023000905	CTGAACACCTCCTGCGGAGG AGG	CGAATATGACAGATCTTATC CGG
chro4_1	4	12411	AO090012000496	AO090012000498	GTAAAACGTTAAAGAGGGCT GGG	ACAATTAGAGATTTATACTG TGG
chro4_2	4	5154	AO090012000820	AO090012000821	AAGCTAAGTATATCGCCTAA TGG	GCTGCTAGTAAAGGTACGGA AGG
chro5_1	5	4859	AO090701000474	AO090701000473	CAATCGAAGAGACAGGAGAA AGG	TATCTTTCATACAACTGTGC TGG
chro6_1	6	5098	AO090038000288	AO090038000286	CAGAGGTACTGCTAGACCTA AGG	AGCACAGTATACGTGTACAC AGG
chro6_2	6	6631	AO090020000403	AO090020000400	TGACATAACGACTCTGGGTT TGG	CTCCTGCACCCTGAACGGGG GGG
chro7_1	7	4873	AO090011000900	AO090011000901	ATTGAAACTCAAGATTAGCT GGG	ACCATCACTACTACAGTTAG TGG
amyA (AO090003001591)	2	N/A			GCACAGATCTGATTAAATCG AGG	ATACTGCCTATTTGTGTCGG TGG

Supplementary Table 5. Targeting plasmids for neutral loci. Our efforts identifying and evaluating neutral loci generated a set of plasmids for straightforward targeting of these loci for gene insertion. The plasmids were cloned in *E. coli* but were then PCR amplified to generate linear fixing templates for *A. oryzae* transformation. The plasmids all have the general format of 5'-homology arm_pTEF1_GFP_TamyB_300bp-loopoutsqeuence_pyrG_3'-homology arm. They are made available through the Joint Bioenergy Institute (JBEI) registry (http://registry.jbei.org). The plasmids for chro3_1 and chro4_2 were not included as they did not show any evidence of integration by PCR (chro3-1) or did not express GFP from the locus (chro4_2).

**Locus targeting plasmid**	**JBx number in JBEI registry**	***E. coli* antibiotic resistance**
chro1_3	JBx_236269	Ampicillin
chro1_2	JBx_236226	Ampicillin
chro2_2	JBx_236270	Ampicillin
chro4_1	JBx_250916	Ampicillin
chro5_1	JBx_236227	Ampicillin
chro6_1	JBx_236228	Ampicillin
chro6_2	JBx_236229	Ampicillin
chro7_1	JBx_236230	Ampicillin

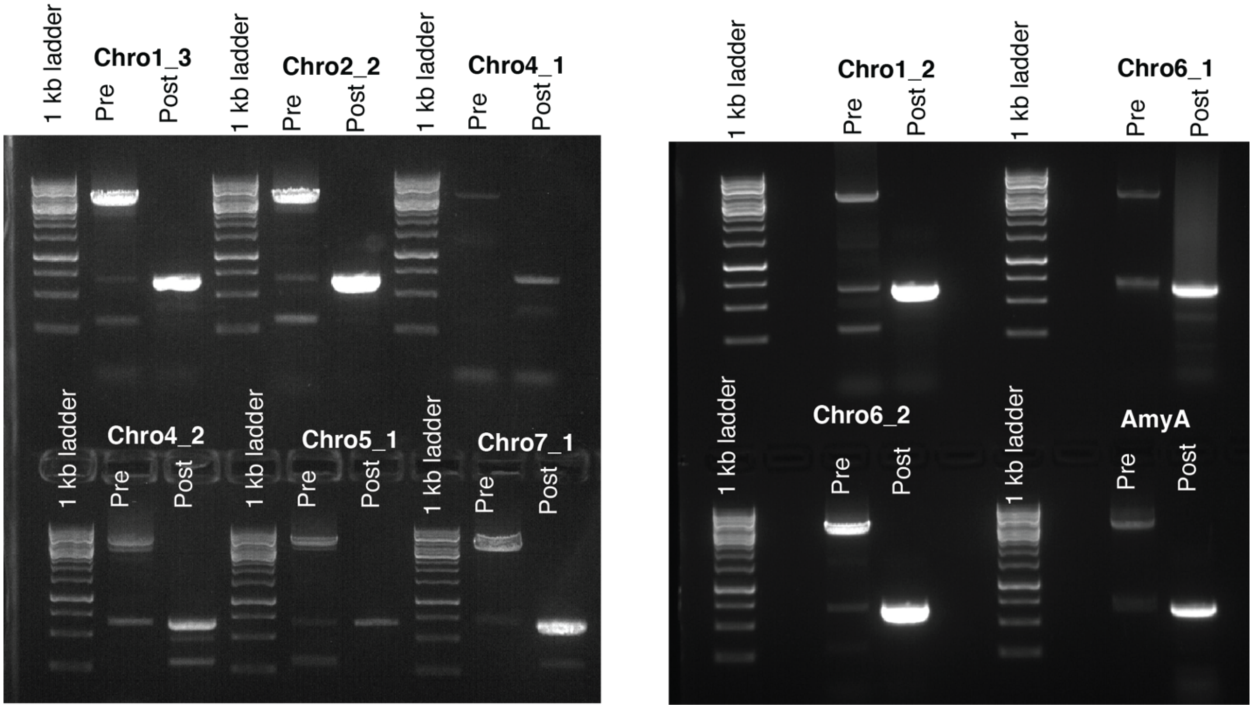

Supplementary Figure 7. PCR confirmation of integration and marker recycling across neutral loci. Locus-specific primers were used to confirm gene insertion and marker recycling from the neutral loci. The general strategy for strain construction is described in Fig. 2 of the main text. The general integration and PCR amplification strategy was based on that for the *wA* locus (Fig. 1C in main text), producing a ~4 kb amplicon for the integrated cassette with the *pyrG* marker intact, and a ~750 bp amplicon for the looped out strain. The ladder is the Generuler 1kb ladder (Thermo Scientific).

A

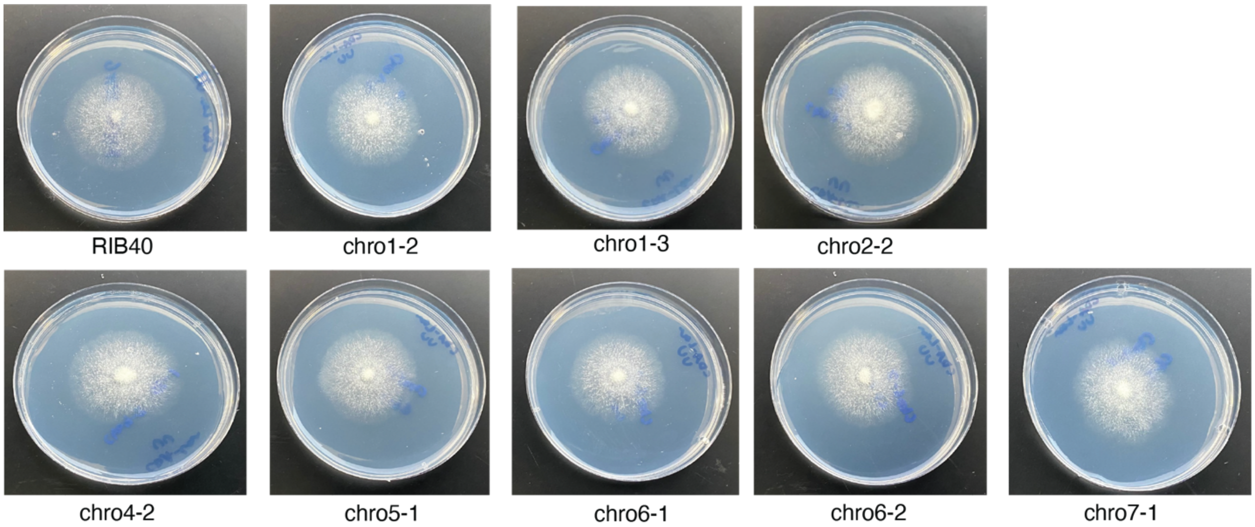

B

Strain	Conidial count (*10^6/mL)
RIB40 ΔpyrG	5.25
chro1_2	10
chro1_3	5.25
chro2_2	6.125
chro4_2	6.5
chro5_1	6
chro6_1	7
chro6_2	5.5
chro7_1	6.5
AmyA control	8.5

Supplementary Figure 8. Colony morphology and conidial counts of looped out transformants harboring expression cassettes neutral loci. A) To explore potential effects of neutral locus integration and gene expression on colony morphology and growth, PCR-confirmed marker recycled stains were grown on CDA medium supplemented with uracil and uridine. There was no gross effect on growth or overall morphology compared to the background RIB40 strain. B) Additionally, we harvested conidia from slants prior to flow cytometry and counted the yield using a hemocytometer. The conidial counts were further used as a proxy for healthy fungal growth[3]. There was no decrease in conidial counts over the background RIB40 strain, further supporting that there were no gross effects of gene integration on fungal physiology.

Supplementary Table 6. Sequences and names of selected core promoters used in Synthetic Expression System evaluation in *A. oryzae*. The ID of the gene that the full-length promoter normally controls in *A. oryzae* RIB40 is shown (ID refers to identifier in the Comprehensive *Aspergillus oryzae* genome database, CAoGD). Full-length pTEF1 was included as a control in the flow cytometry screen.

**Core promoter name**	**Core promoter sequence**	**RIB40 gene ID (CAoGD)**	**Reference (if applicable)**
An_201205	TTCTCTTTTCTTAAGAATATGTTCAAAGACTAGGATGGATAA<br>ATGGGGTATATAAAGCACCCTGACTCCCTTCCTCCAAGTTC<br>TATCTAACCAGCCATCCTACACTCTACATATCCACACCAAT<br>CTACTACAATTATTAATTAAA		SES in *T. reesei* and *A. niger*[4]
AFL_ecm33	GTTGCTATGCATTCGGGGTATAGCCGTGATTGGCTCAAGA<br>TTGGCCTACTAGATCCACCTTTCTCGCCATTGGGACACAAT<br>CAATTCTCATCTCAAGAGTCCATAACGCCATCATTGAGGAT<br>ATGGAAATGGGGTATAGTAAGGATTAAGTAAGATACATTGC<br>ATAAAGGATGGCAATTCACTTTTTCTGTACGGGGGAT		This study (full-length promoter is from *A. flavus* from gene AFLA_113120 / promoter P4 in a previous study[5]
Ao_thiA	GCATTCACATGCCCTCCCTAAGCTGGGCCCTAGACTCTAG<br>GATCCTAGTCTAGAAGGACATGGCATCGATGGACTGGGTT<br>CGTTCTGAGATTATACGGCTAAAACTTGATCTGGATAATAC<br>CAGCGAAAAGGATCATGCCTTCTCTCGTTCTTCCTGTTGAT<br>GGAATGGCTAACAGATGATAGTCATTGCAACTTGAAAC	AO090003000090	This study
Ao_tef1	TTCCTACTCTTCTTCCATTCATTCAACTCTTCTCCTTACTGA<br>CATCTGTTTTGCTCAGTACCTCTACGCGATCAGCCGTAGTA<br>TCTGAGCAAGCTTCTCTACAGAATCTTTCTAGTATCTTACAA<br>AGAACTACAAAGTTCGCACCACCTTCAAAATGGGGTAAGTT<br>TATCAACCCGTCGAGTTGTGTTGCATCTCAGATC	AO090120000080	This study
Ao_aspnd1	GATACTCCCGTACGATCGCCCGCAGCTATACCGAAATCTC<br>TGAATACGATCCGTGGAGGCACCTTGGCAACAGCCCACGA<br>CAAGAAGAGACAAGCAATCCACGTCCAGAAACCGCAGTAT<br>AAAAGCCCATCTAGAACCCTGGCCATGTAGATGCATAGACT<br>TCACAGACCTCCCAAGTATCTTTTCAAAGCCTATCCACC	AO090005000939	This study
Ao_amy1	TGCAGAATGCAATTTAAACTCTTCTGCGAATCGCTTGGATT<br>CCCCGCCCCTGGCCGTAGAGCTTAAAGTATGTCCCTTGTC<br>GATGCGATGTATCACAACATATAAATACTAGCAAGGGATGC<br>CATGCTTGGAGGATAGCAACCGACAACATCACATCAAGCT<br>CTCCCTTCTCTGAACAATAAACCCCACAGAAGGCATTT	AO090023000944	This study
Ao_0336	CATGCGGGTGGGTCATAGCCTTTGACACTGCAGCAGTCTA<br>GCTTGTCAAAGTCAGATGTAAGAGATTTCATCAAAGGAGGG<br>GTTAATGCACTCTGCAGGGGTATAACTTAGGGGTTGTCCCT<br>CCTTGCGTTCCTTTTGTATTATAGGTCTACTTGTAGACGTGT<br>CTCTCATTCTTGGTACCATCCACTCAAGTCAGAAAG	AO090011000336	This study
Ao_0666	TTTGATACAGCCCGTGGACTGTGATTTATGTCGGCTAATTC<br>TGACCGGCTGGTCAAGACAAATTAAGAGCGGAGTTACCTT<br>GGAGGCCGCTACTAGGGTGGAGGTATAAAGCCAATCCGAT<br>TTCCCTTGTTGTGCCTTCTCATCCCGCCACTTCCAAACGCA<br>CATAAGCTCGGACTATATCTCGCACAACTGCAAGAACC	AO090010000666	This study
Ao_prxA	TAGGTGTGCCTCTTACGGCCCGTGGGACGCGATCTCCGAG<br>AGGGAATCAACCCATATATTAACCGCCACATGCTCCTGCCC<br>CTCGCTCCTACCTTCTGACTTTCCACATCACCCACATCACA<br>TAAATAAACACCTTTTTCCATCCACAACCAACTATTCTTTTC<br>ACTCCCAACCCAACCTAACATACCAATTCAATCAAA	AO090120000112	This study
Ao_hhfA	GGTCGATCCTCTTGCTAGCGCGTGATTCCAACGCCCTCCC<br>CGCGCTCTTCCTTTTCCTATACAAATATCACCCGTCCCATG<br>GCAGCTGTCCCAATCTTTCCCTTCCCATCTCTCTCACATCT<br>GTCATCCAATTAACCACCTATCTCTCCACATTTTCCCACTCA<br>ACCTTAAACAAGTTAACTCTTTAATCAACCATCAAT	AO090012000496	This study
Ao_pdcA	CATGACATCACGTTATCATGACGTATGGCCTGGTCTTACTG<br>TTGCACGTCGAGTTGTACAGGACATTCTGTATTCACTACTA<br>TATAACTGCCCCCCCTTGTCCATGTTTCCAGGAAGAATTCA<br>GTATCCAACAATCCATAACAACAACCTTATCATCTACTATAC<br>CTTCCATTTTCCATTCCTCTTACTGAGTCCTTACA	AO090003000661	This study
Ao_gpdA	CCGTCCGACTTTTCTCCCCTTTTCTACTCTCTTCGTATACTC<br>ACCACTGCAATCACCTTATCCCTTTGTCTTTTTACTTAAAGT<br>GAGTCGTCTCCCGCCCATCATTCCCTTTGGATCTTCACTTT<br>CAAGTGCCTACCGTTTCCCTTTCCACACAGATTGACTGACA<br>GCTACCCCGCCACACCAACAGACACATCTAAACA	AO090003001322	This study

Ao_0583	TATATAAGGGATTGGAGCATGGATGGACTTGGTGTCTATTC<br>TTCATCATCAGAACAACATAACAAAGCAAGCAAGTCTATCT<br>TACAGCAACTACTATCTTGTTCACTGCGCTGAATAACCTCT<br>TGGGTTCATCGAATTGCCCTACTCTATCCTCTACTATCCAT<br>CAACCGAATCCATCGGACAAAAACCCACAAGGCAAC	AO090005000583	This study
pTEF1, full length		AO090120000080	This study

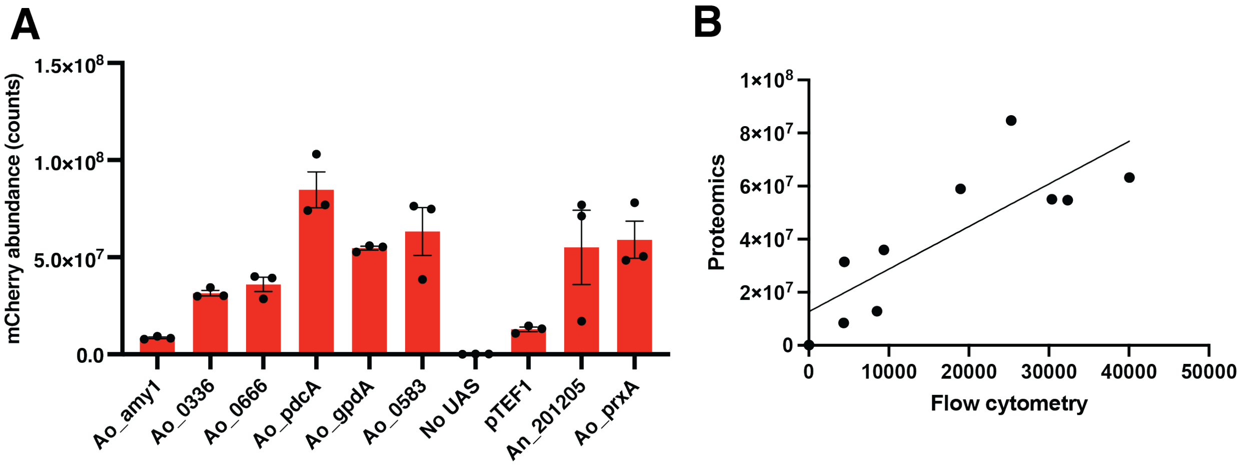

Supplementary Figure 9. Evaluation of core promoter strength in mycelia. A) Proteomics was used to evaluate the expression levels of mCherry in mycelia (grown in liquid culture in CD-glucose medium) from a subset of the core promoters in the SES. This analysis highlighted that, similar to the results seen using flow cytometry in conidia (Fig. 3 of main text), the core promoter can drive gene expression across different strengths, including at levels several-fold higher than the constitutive promoter pTEF1. Results are from three biological replicates and represent the mean and SEM. B) Correlation between conidia flow cytometry data (METR, from Fig. 3D in main text) and mycelial proteomics data (mCherry abundance, from A). Individual data points represent the mean expression level from a particular promoter. The line represents the best-fit trendline for linear regression. There was a statistically significant correlation between the mean expression levels identified using each method (n=10 pairs, r=0.82, R^2=0.67, p=0.0039), suggesting that flow cytometry is a useful screening approach to identify constitutive promoters that are also active mycelia. The statistical test for correlation was conducted using Graphpad Prism (v.10.1.1).

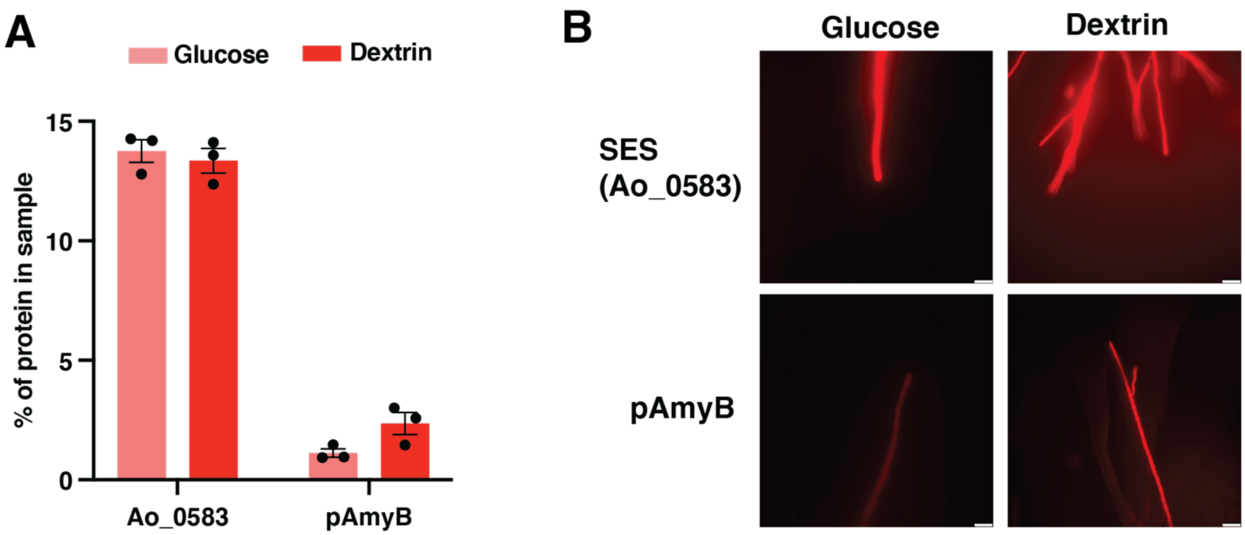

Supplementary Figure 10. Microscopy and intracellular protein comparison across media for the synthetic expression system and the commonly used promoter pAmyB. A) Using the top3 peptide method for protein abundance estimation in proteomics data, the intracellular content of mCherry across glucose and dextrin media, for both the synthetic expression system (SES) and pAmyB, was calculated. Results are average and SEM of three biological replicates. B) Imaging of SES and pAmyB strains grown across carbon sources. Strains were grown on CDA medium with either dextrin or glucose as the carbon source prior to imaging by fluorescence microscopy. Scale bar = 25 µm for glucose, 50 µm for dextrin.

Supplementary Table 7. ***A. oryzae*** **endogenous bidirectional promoter candidates selected for experimental evaluation.** Sequences were identified using a computational approach (see Supplementary Figure 10 for overall approach). The flanking genes are shown (gene name for *A. oryzae* RIB40). The 5' and 3' gene ID refers to identifier in the Comprehensive *Aspergillus oryzae* genome database, CAoGD.

Bidirectional promoter name	Bidirectional promoter size (bp)	5' gene	3' gene
p2-1	1439	AO090003000055	AO090003000056
p4-1	1881	AO090012000994	AO090012000995
p4-2	821	AO090012000495	AO090012000496
p6-1	1596	AO090020000518	AO090020000517
p7-1	743	AO090011000414	AO090011000415

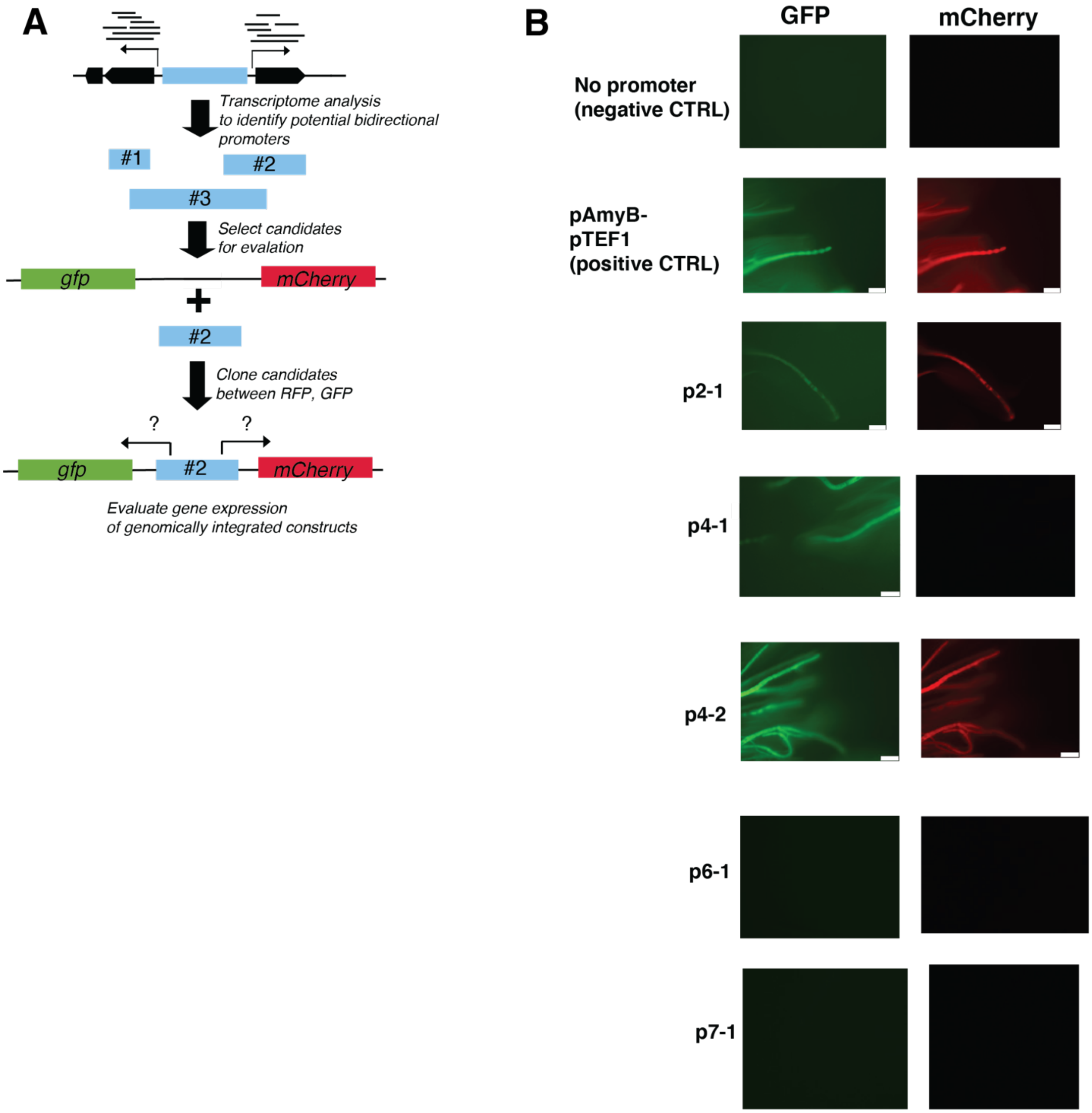

Supplementary Figure 11. Evaluation of potential endogenous bidirectional promoters in *A. oryzae.* A) Strategy for identification of candidate endogenous bidirectional promoters. Promoter candidates were considered as intergenic regions between co-localized genes displaying high levels of expressions and pointing in opposite directions. To evaluate their potential for driving bidirectional expression, promoter candidates were cloned between mCherry and GFP B) Imaging of bidirectional promoter candidates. Two candidates (p2-1 and p4-2) demonstrated bidirectional

promoter characteristics. A concatenated sequence of pAmyB and pTEF1 pointing in opposite directions was included as a positive control to validate the design. Scale bar = 25 μm.

Supplementary Table 8. pBLAST results for *N. crassa* ergothioneine biosynthetic enzymes Egt1 and Egt2 in *A. oryzae* RIB40. Candidate ergothioneine biosynthetic enzymes in *A. oryzae* were identified by a pBLAST search in UniProt using the characterized *N. crassa* enzymes[6,7] as the query.

***N. crassa* enzyme**	***N. crassa* UNIPROT ID**	***A. oryzae* top hit (UNIPROT ID)**	***A. oryzae* top hit (gene ID)**	**% ID**	**Length (AA)**	**E-value**
**Egt-1**	Q7RX33	Q2UD95	AO090012000265	49.2	845	0
**Egt-2**	A7UX13	Q2UF99	AO090026000291	45.1	461	7.10E-127

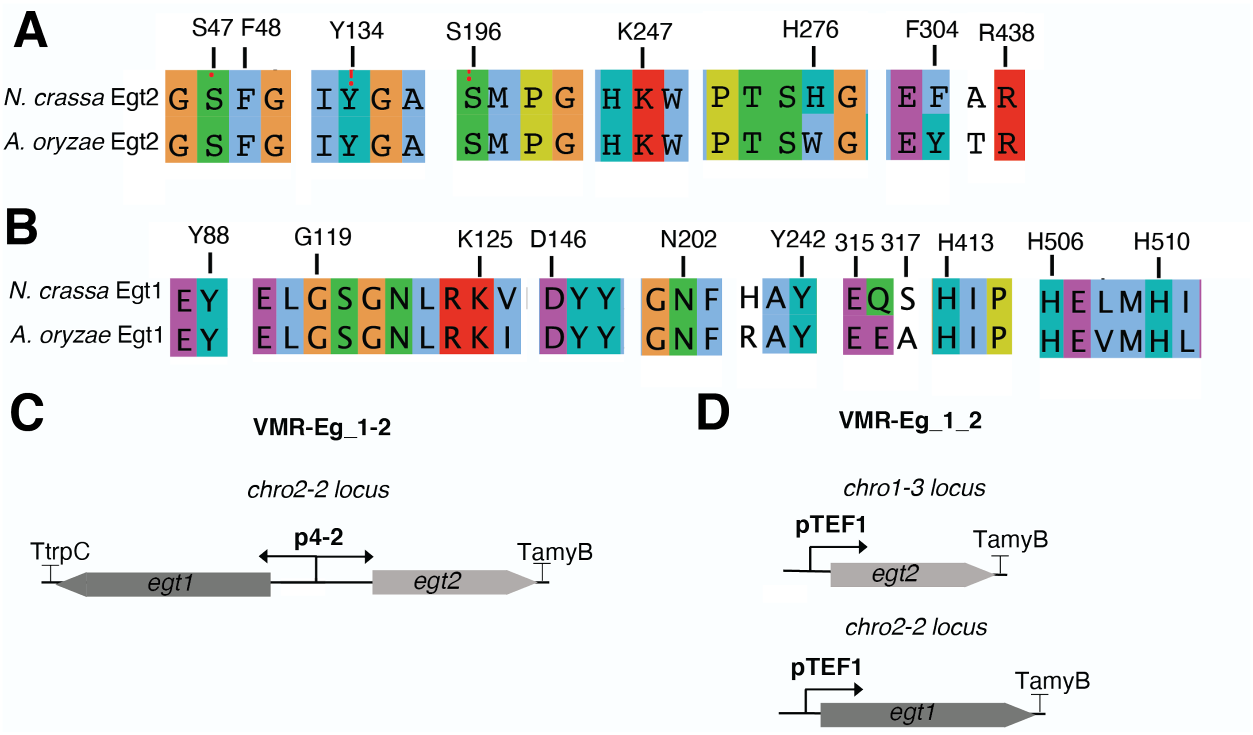

Supplementary Figure 12. Sequence alignment of *N. crassa* Egt1 and Egt2 and *A. oryzae* homologs and engineering strategy for enhancing ergothioneine biosynthesis in *A. oryzae*. A) Alignment of Egt2 homologs. The *N. crassa* enzyme (Uniprot ID A7UX13) has been structurally characterized[7]. Active site residues are shown. K247 is involved in binding of PLP, the essential cofactor. The remaining residues surround the substrate in the binding pocket. H276 only loosely associates with the histidine moiety of the substrate through potential hydrogen bonding with the imidazole ring. Thus, it is predicted that the corresponding W residue in *A. oryzae* Egt2 could make similar contacts. Similarly, the *A. oryzae* Y in the position of the *N. crassa* F304 is predicted to participate in the distant cation-π interaction between the trimethylamino group of the substrate and the aromatic ring that is seen in the *N. crassa* enzyme. B) Unlike Egt2, Egt1 has not been structurally characterized. Instead, residues bioinformatically predicted to be involved in enzyme function are shown. Y88, Y242, E315, Q316, and S318 are predicted to be involved in histidine binding, while H413, H506, H510 are predicted to bind iron in the non-heme iron enzyme. Finally,

G119, K125, D146 are predicted to bind SAM. Sequence alignments were generated using CLUSTAL in Snapgene (v. 10.1.1). Alignments were inspected for residues structurally confirmed or bioinformatically predicted to be important for substrate binding or catalysis. C) and D) the *A. oryzae* Egt1 and Egt2 homologs were integrated at neutral loci into *A. oryzae*, either under the control of the p4-2 bidirectional promoter, or as separately integrated genes driven by the constitutive pTEF1 promoter at two distinct neutral loci, giving rise to strains VMR-Eg1-2 and VMR-Eg1_2, respectively.

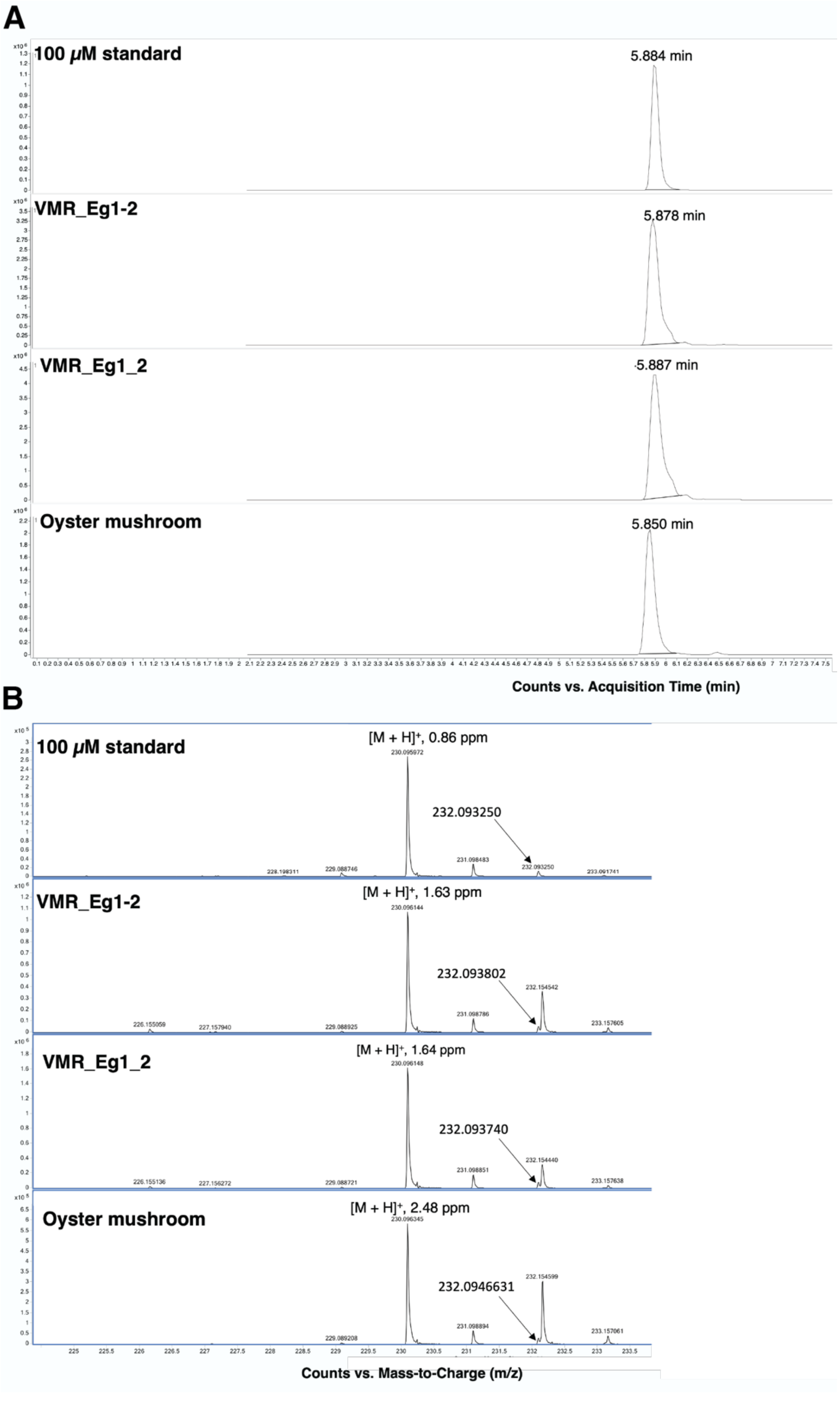
A
100 μM standard
5.884 min
VMR_Eg1-2
5.878 min
VMR_Eg1_2
5.887 min
Oyster mushroom
5.850 min
Counts vs. Acquisition Time (min)
B
100 μM standard
[M + H]+, 0.86 ppm
230.095972
232.093250
VMR_Eg1-2
[M + H]+, 1.63 ppm
230.096144
232.093802
232.154542
VMR_Eg1_2
[M + H]+, 1.64 ppm
230.096148
232.093740
232.154440
Oyster mushroom
[M + H]+, 2.48 ppm
230.096345
232.0946631
232.154599
Counts vs. Mass-to-Charge (m/z)

Supplementary Figure 13. Detection of ergothioneine using high-resolution liquid chromatography-mass spectrometry. Extracted ion chromatograms and mass spectra for ergothioneine across different samples are shown. Hydrophilic interaction liquid chromatography was coupled to high-resolution accurate mass spectrometry via ESI in the positive ion mode to obtain extracted ion chromatograms for ergothioneine (refer to "*Extraction and LC-MS analysis of ergothioneine and heme in fungal mycelium and reference samples*" in the methods section). A) The retention time of ergothioneine in samples were consistent with that of the chemical standard (at a concentration of 100 μM). B) The observed mass spectra from 224.5 to 233.5 *m/z* revealed the monoisotopic $[M + H]^+$ ergothioneine metabolite and chemical standard ions were ~230.096 *m/z* with good mass accuracy and low mass error at < 2.5 ppm. This peak at 230 *m/z* has been previously identified as the major species in LC-MS[8]. Additionally, the carbon isotopic distribution patterns for ergothioneine in the samples were consistent with that of the chemical standard. The third isotope peak is highlighted with the arrow. These data provide confirmation of ergothioneine identification in the samples. The raw data (output from Masshunter software) is presented in Supplementary data file 3.

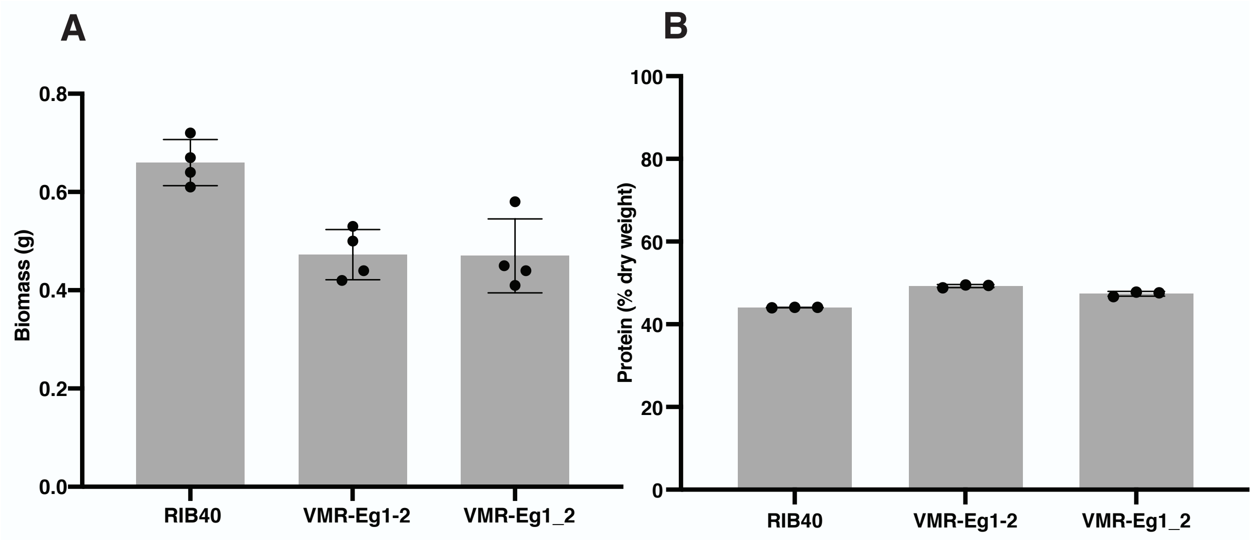

Supplementary Figure 14. Growth yield and protein content and of engineered ergothioneine strains. A) Dry biomass yield of strains. There was a reduction in growth in the engineered strains relative to the wild-type RIB40 strains. The harvested fungal biomass was subjected to drying to remove all moisture prior to being weighed. Results are mean and SEM of four biological replicates. Strains were grown for 96 hours in GP-glucose medium. B) Protein content in the strains after 96 hours of growth. The protein content did not differ majorly between strains. Results are mean and SEM of three biological replicates.

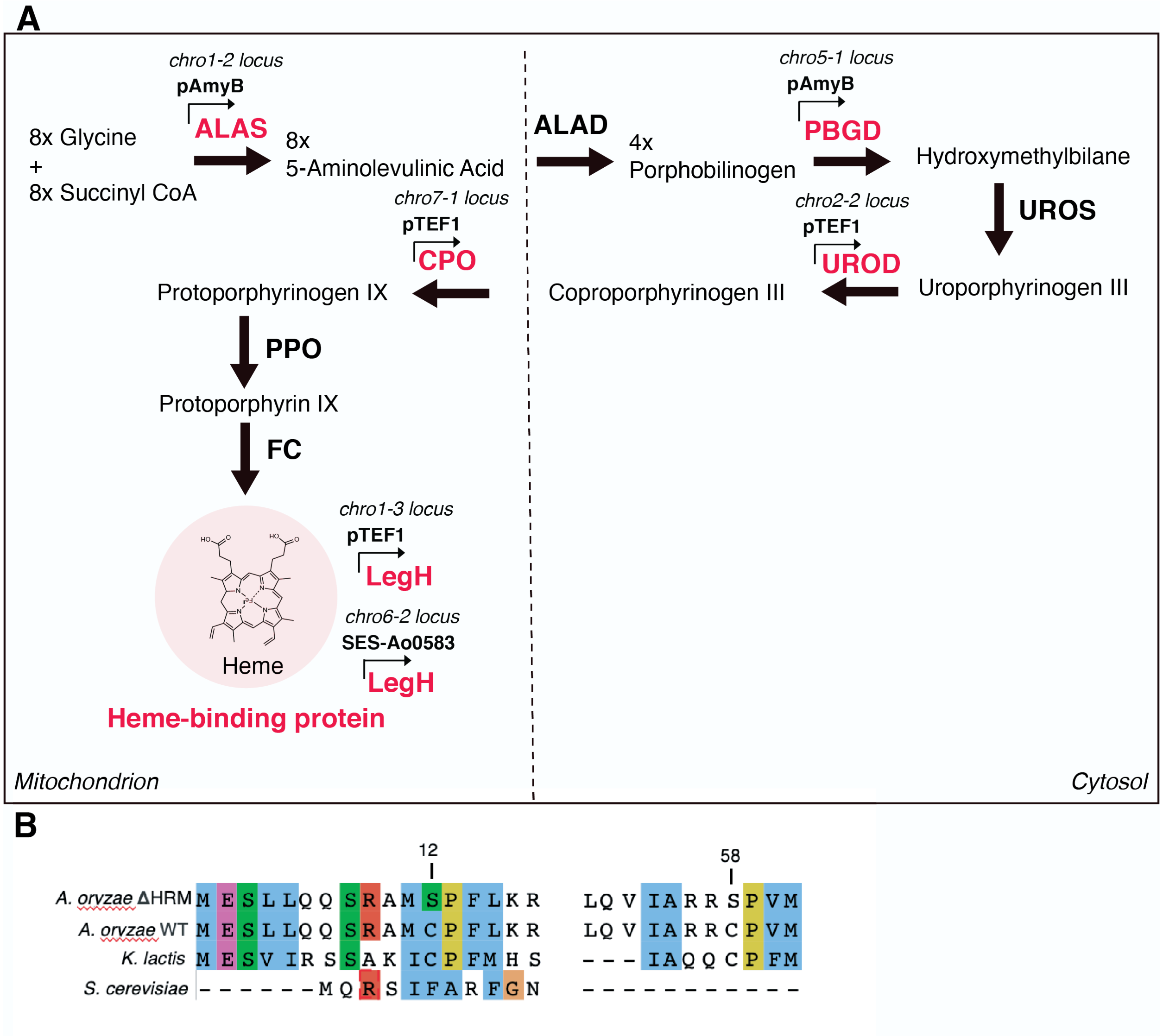

Supplementary Figure 15. Heme biosynthesis pathway and initial engineering strategy. A) Heme biosynthetic pathway in fungi. The complex pathway includes 8 dedicated enzymes, which are split between the mitochondrion and the cytosol. Engineering targets are highlighted in red; arrows show the promoter that was used and the text above indicates the neutral locus at which the construct was integrated. Two copies of the FDA-approved soy leghemoglobin LegH, used in IMPOSSIBLE foods, was included as a heme-binding protein to act as a potential sink for heme, to minimize cytotoxicity and potentially drive pathway flux[9-11]. Whereas one copy was expressed using the recently discovered SES (the Ao_0583 promoter), the other copy was expressed using

the significantly weaker endogenous pTEF1 promoter. B) Additionally, we mutated key cysteine residues in the Heme Regulatory Motif (HRM) of ALAS to remove potential feedback inhibition by heme. Alignment of *A. oryzae* ALAS with *K. lactis* and *S. cerevisiae* sequences. Unlike *A. oryzae* and *K. lactis, S. cerevisiae* ALAS does not have a HRM. The two predicted cysteine residues in the *A. oryzae* HRM were mutated to serine residues.

Supplementary Table 9. Predicted heme biosynthesis enzymes in *A. oryzae*. Candidate enzymes were identified by a pBLAST search in Uniprot using the corresponding characterized *S. cerevisiae* enzyme as the query. The *A. oryzae* genome tag is from the RIB40 genome in the Comprehensive *Aspergillus oryzae* genome database, CAoGD

***S. cerevisiae* enzyme**	***S. cerevisiae* UNIPROT ID**	***A. oryzae* top hit, UNIPROT ID**	***A. oryzae* top hit, genome tag**	**% ID**	**Length (AA)**	**E-value**
5-aminolevulinate synthase, mitochondrial	P09950	Q2UTR0	AO090009000630	56.1	636	0
Delta-aminolevulinic acid dehydratase	P05373	Q2UPG4	AO090005001652	66.2	375	1.1E-161
Porphobilinogen deaminase	P28789	Q2TYQ6	AO090103000151	49.2	333	4.30E-103
Uroporphyrinogen-III synthase	P06174	Q2ULI2	AO090003000401	33.1	309	8.70E-27
Uroporphyrinogen decarboxylase	P32347	Q2UKH8	AO090003000803	58.9	378	2.70E-151
Oxygen-dependent coproporphyrinogen-III oxidase	P11353	Q2UBH4	AO090012000998	52.9	437	6.80E-122
Protoporphyrinogen oxidase	P40012	Q2UN54	AO090001000500	25.9	597	7.70E-38
Ferrochelatase, mitochondrial	P16622	Q2U7V9	AO090701000682	56.7	424	1.90E-133

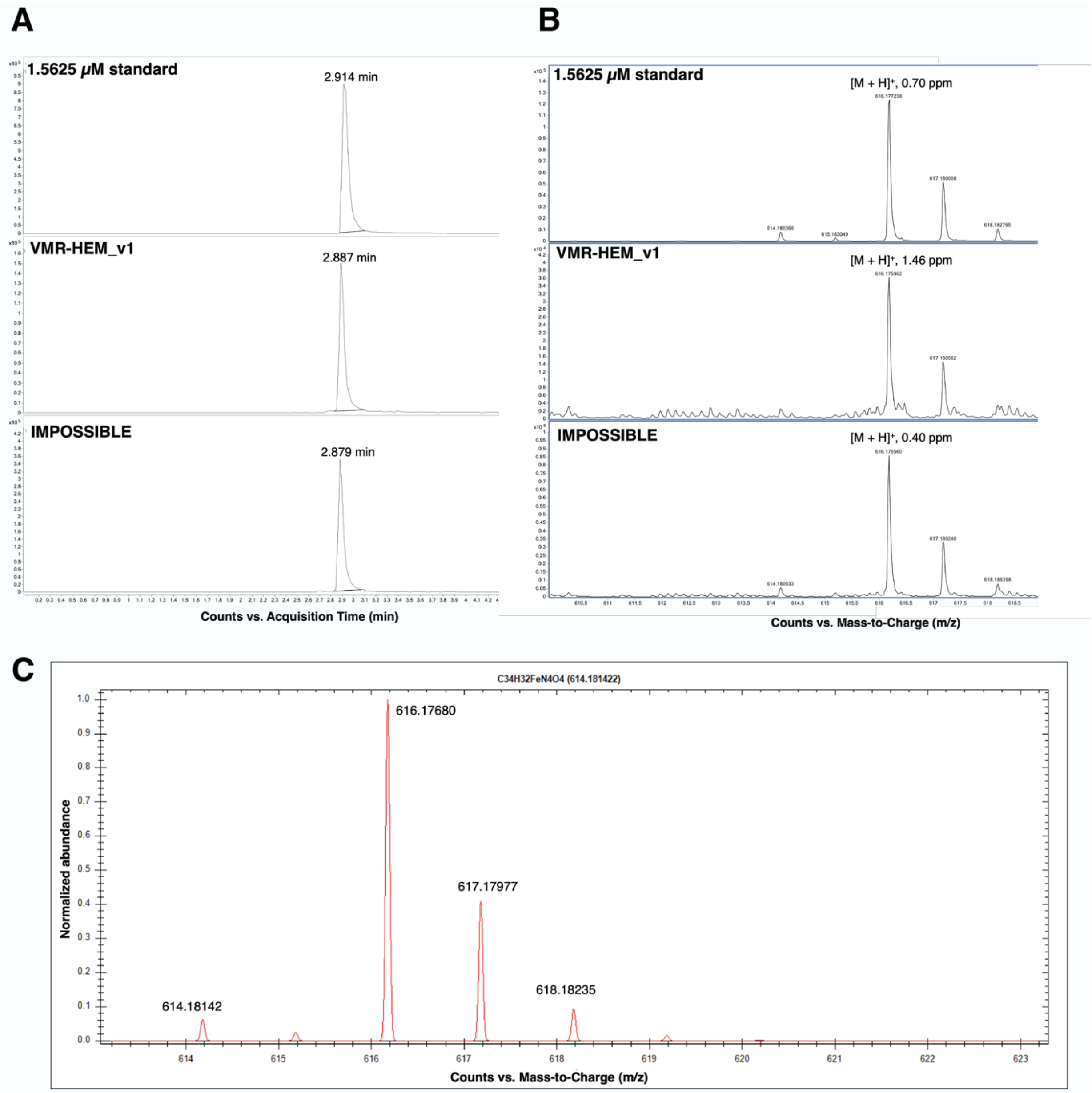

Supplementary Figure 16. Detection of heme using high-resolution liquid chromatography-mass spectrometry. Reversed-phase liquid chromatography separation was coupled to high-resolution accurate mass spectrometry via ESI in the positive ion mode to obtain extracted ion chromatograms for heme (refer to method section *"Extraction and LC-MS analysis of ergothioneine and heme in fungal mycelium and reference samples"*). A) The retention time of heme in samples was consistent with that of the chemical standard (at a concentration of 1.5625 μM). B) The observed mass spectra from 610 to 624 *m/z* revealed the monoisotopic M^+ heme

metabolite and chemical standard ions to be ~616.177 *m/z* with good mass accuracy and low mass error at < 1.5 ppm. C) The calculated isotope pattern for the heme molecular formula $C_{34}H_{32}N_4O_4Fe$ (by the Agilent Technologies Isotope Distribution Calculator software). The carbon isotopic distribution patterns for heme in the samples were consistent with that of the chemical standard and theoretical calculation, as well as the experimentally observed species in previous studies of cellular heme[12]. This data provides confirmation of heme identification in samples. The raw data (output from Masshunter software) is presented in Supplementary data file 4.

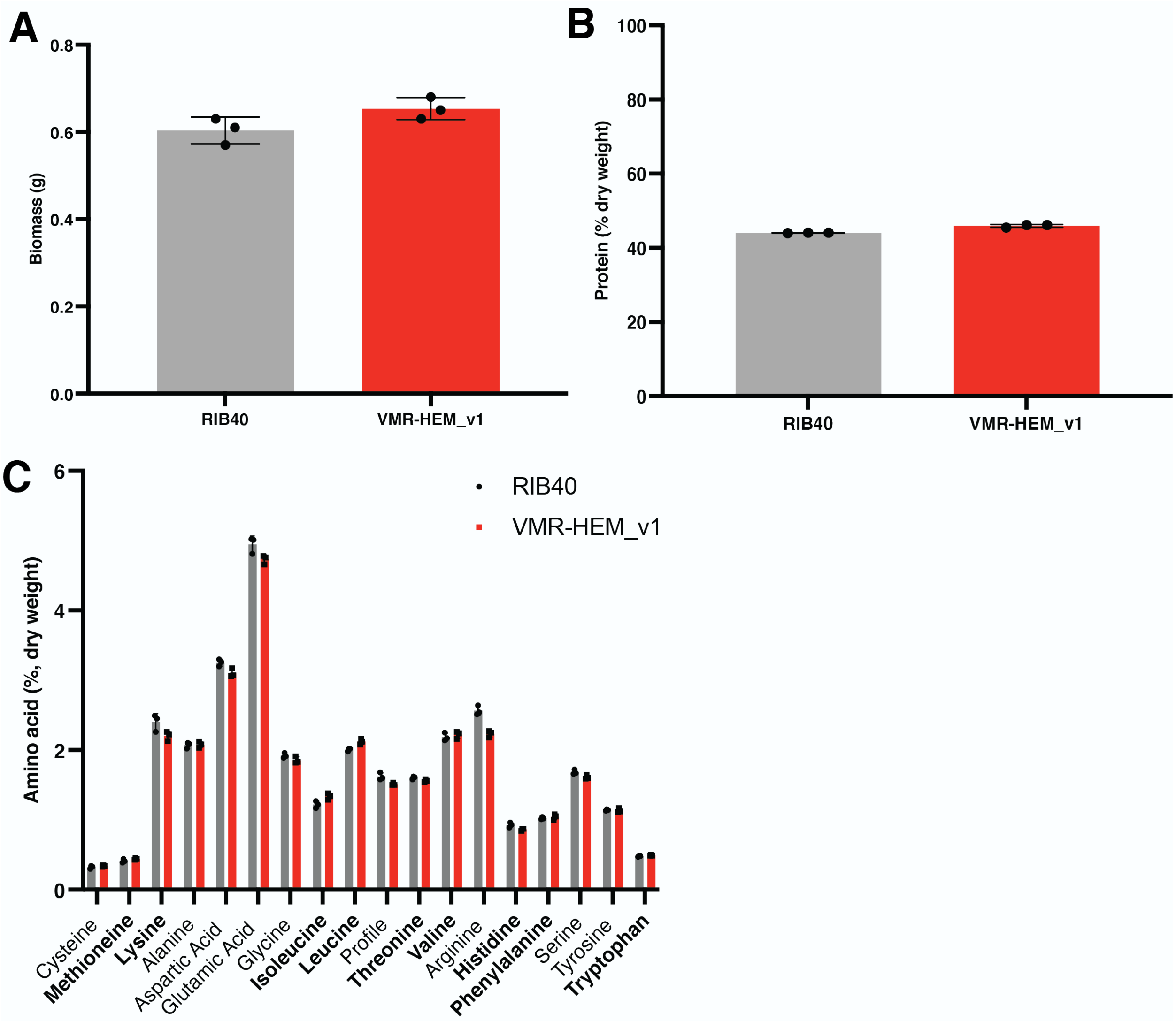

Supplementary Figure 17. Growth yield and nutritional analysis of wild-type biomass and biomass from the strain engineered for elevated heme production. A) Dry biomass yield of strains. There was no significant difference in growth between the two strains. The harvested fungal biomass was subjected to drying to remove all moisture prior to being weighed. Results are mean and SEM of three biological replicates. B) Protein content in the strains after 96 hours of growth. The protein content (~45%) was similar between the two strains. Results are mean and SEM of three biological replicates. C) Total amino acid composition in wild-type and engineered biomass. Lyophilized biomass was subjected to amino acid composition analysis after 96 hours of

growth. There was no major change in amino acid composition in the engineered strain, which also contained all 9 essential amino acids (highlighted in bold). Results are mean and SEM of three biological replicates.

Supplementary Table 10. Primers used for genome modification and confirmation in this study.

Primer name	Primer sequence (5'->3')	Explanation
TamyB-DC-F	AGGGTGGAGAGTATATGATGG	Binds to TamyB terminator, used to verify insertion / excision
AmyA_DC-f_F	CGTAACAGGATAGCCTAGAC	PCR linearization of AmyA fixing template for transformation
AmyA_DC-f_R	GATAGCATGATTGGGGGATATA	PCR linearization of AmyA fixing template for transformation
amyA_in_1R	GGTTCCAAGCGGAGTCTGAG	With TamyB-DC-F, confirm insertion / excision from amyA locus
niaD_DC-f_F	CGCGGAGATGGTGCAAAGTT	PCR linearization of niaD fixing template for transformation
niaD_DC-f_R	GTTCCCGTTTTCCTTCGTAATTG	PCR linearization of niaD fixing template for transformation
niaD_in_1R	CGTTCCAATATGGTACTCAGG	With TamyB-DC-F, confirm insertion / excision from niaD locus
ya_DC-f_F	CGGGTCCATCTGCATTCTG	PCR linearization of yA fixing template for transformation
ya_DC-f_R	ATGTGGTCGGATCCGAGGA	PCR linearization of yA fixing template for transformation
ya_in_1R	AATCATACATCGACCGCCTC	With TamyB-DC-F, confirm insertion / excision from yA locus
wa_DC-f_F	ATGGTTTATCGTTCGGATAG	PCR linearization of wA fixing template for transformation (950 bp homology arms)
wa_DC-f_R	GAACTTGAAATAGTAGTTTATCC	PCR linearization of wA fixing template for transformation (950 bp homology arms)
wa_DC_250-1F	AGGAGCTTCGGAAACTGAGT	PCR linearization of wA fixing template for transformation (250 bp homology arms)
wa_DC_250-1R	TGACGTAGTAGAATCTCTTCAAG	PCR linearization of wA fixing template for transformation (250 bp homology arms)
wa_DC_100-1F	CAGCGGTCTCAAGAGCGTAA	PCR linearization of wA fixing template for transformation (100 bp homology arms)
wa_DC_100-1R	ACATCTCGGCCATAGTACAG	PCR linearization of wA fixing template for transformation (100 bp homology arms)
wa_DC_25-1F	CCCAATGGAGACGATCGTTA	PCR linearization of wA fixing template for transformation (25 bp homology arms)
wa_DC_25-1R	GACAGCCTAGAATCAATTAAATCA	PCR linearization of wA fixing template for transformation (25 bp homology arms)
wa_in_1R	AGCTTGAGTACCAGTTCCAT	With TamyB-DC-F, confirm insertion / excision from wA locus
wa_in_1F	TTTTAGCGGAACCGCTCTTT	With pAmyB-3R, colony PCR for confirmation of insertion at *wA* locus
pAmyB-3R	TATCAGCCCTAACGTAATCGG	With wA-in-1F, colony PCR for confirmation of insertion at *wA* locus
pyrG-2F	AGGTGTAGCACCAGTGGCTAT	With pyrG-2R, amplify *pyrG* gene and surrounding region to assess mutation in *pyrG* gene

pyrG-2R	CTAGACAGCTTTGGAGCGGATA	With pyrG-2R, amplify *pyrG* gene and surrounding region to assess mutation in *pyrG* gene
chro1_2_lin-2F	TTGGTTTGTATATGTGCTAATC	PCR linearization of chro1-2 fixing template for transformation at neutral locus
chro1_2_lin-2R	TCACCGATCACAAATTCTAGT	PCR linearization of chro1-2 fixing template for transformation at neutral locus
chro1_2_DC-R	TTGCCAATGCTGTCACGTCAG	With TamyB-DC-F, confirm insertion / excision from chro1-2 locus
chro1_3_lin-F	TCTAGAGAATGTTGATCTCAAG	PCR linearization of chro1-3 fixing template for transformation at neutral locus
chro1_3_lin-R	GGCCGATGTTAAATTTAGCA	PCR linearization of chro1-3 fixing template for transformation at neutral locus
chro1_3_DC-R	ACCCATTTACTTCGTGCTCAT	With TamyB-DC-F, confirm insertion / excision from chro1-3 locus
chro2_2_lin-F	TTTTTTTTCCCTCTTCCCCA	PCR linearization of chro2-2 fixing template for transformation at neutral locus
chro2_2_lin-R	AGTATACACACGCAATTTAAAA	PCR linearization of chro2-2 fixing template for transformation at neutral locus
chro2_2_DC-R	CTTGGTGTTCGACTTCGGGTA	With TamyB-DC-F, confirm insertion / excision from chro2-2 locus
chro4_1_lin-F	AAAAACACACACATACTGAAAT	PCR linearization of chro4-1 fixing template for transformation at neutral locus
chro4_1_lin-R	GTAAGATGTTATGACTCGTCG	PCR linearization of chro4-1 fixing template for transformation at neutral locus
chro4_1_DC-R	TGATCTGATTCAGACTCCATC	With TamyB-DC-F, confirm insertion / excision from chro4-1 locus
chro5_1_lin-F	TATGTTATATCCAACGGTTTCG	PCR linearization of chro5-1 fixing template for transformation at neutral locus
chro5_1_lin-R	AATATCTACCTTGGAGTCACG	PCR linearization of chro5-1 fixing template for transformation at neutral locus
chro5_1_DC-R	GTTATTACATAAAGTCTGCCCT	With TamyB-DC-F, confirm insertion / excision from chro5-1 locus
chro6_1_lin-F	GCTGTACCTAATGGTGCCCA	PCR linearization of chro6-1 fixing template for transformation at neutral locus
chro6_1_lin-R	TATTTCCCTCCCGTAAGGTAC	PCR linearization of chro6-1 fixing template for transformation at neutral locus
chro6_1_DC-R	CAGTACGTTCGAGGCTCAAG	With TamyB-DC-F, confirm insertion / excision from chro6-1 locus
chro6_2_lin-F	TTTTACTATGCGCGATTTTCC	PCR linearization of chro6-2 fixing template for transformation at neutral locus
chro6_2_lin-R	CCGTAGTCTCACTAGACTCCA	PCR linearization of chro6-2 fixing template for transformation at neutral locus
chro6_2_DC-R	CCGCTACTTTTGCTACTACTA	With TamyB-DC-F, confirm insertion / excision from chro6-2 locus
chro7_1_lin-F	CTGGCCACGCCAGATACAAT	PCR linearization of chro7-1 fixing template for transformation at neutral locus
chro7_1_lin-R	TGACACCAAAGGCTACACCTA	PCR linearization of chro7-1 fixing template for transformation at neutral locus
chro7_1_DC-R	TCCCTTTGTAAAACCTGAAAGG	With TamyB-DC-F, confirm insertion / excision from chro7-1 locus

Supplementary Table 11. Plasmids used for genome modification of *A. oryzae*. More information about neutral locus plasmids can be found in Supplementary Table 3.

JBEI Number	Description	Marker	Related Figure	Comment
JBx_250918	*wA*::pAmyB-GFP-TamyB	Amp	Fig. 1, Supplementary Figures 1, 4	*wA* locus targeting plasmid
JBx_250920	*yA*::pAmyB-GFP-TamyB	Amp	Supplementary Figure 5	*yA* locus targeting plasmid
JBx_250922	*niaD*::pAmyB-GFP-TamyB	Amp	Supplementary Figure 5	*niaD* locus targeting plasmid (full, GFP)
JBx_250924	*niaD*::pAmyB-mCherry-TamyB	Amp	Fig. 1	*niaD* locus targeting plasmid (full, mcherry)
JBx_250926	*niaD*::pAmyB-mCherry-TamyB-nopyrG	Amp	Supplementary Figure 6	*niaD* locus targeting plasmid (no pyrG marker)
JBx_250928	*AmyA*::pTEF1-GFP-TamyB	Amp	Fig. 2	*AmyA* locus targeting plasmid
JBx_236269	chro1_3:: pTEF1-GFP-TamyB	Amp	Fig. 2, Supplementary Tables 4-5, Supplementary Figure 7	Chro1_3 neutral locus targeting plasmid
JBx_236226	chro1_2:: pTEF1-GFP-TamyB	Amp	Fig. 2, Supplementary Tables 4-5, Supplementary Figure 7	Chro1_2 neutral locus targeting plasmid
JBx_236270	chro2_2:: pTEF1-GFP-TamyB	Amp	Fig. 2, Supplementary Tables 4-5, Supplementary Figure 7	Chro2_2 neutral locus targeting plasmid
JBx_250916	chro4_1:: pTEF1-GFP-TamyB	Amp	Fig. 2, Supplementary Tables 4-5, Supplementary Figure 7	Chro4_1 neutral locus targeting plasmid
JBx_236227	chro5_1:: pTEF1-GFP-TamyB	Amp	Fig. 2, Supplementary Tables 4-5, Supplementary Figure 7	Chro5_1 neutral locus targeting plasmid
JBx_236228	chro6_1:: pTEF1-GFP-TamyB	Amp	Fig. 2, Supplementary Tables 4-5, Supplementary Figure 7	Chro6_1 neutral locus targeting plasmid
JBx_236229	chro6_2:: pTEF1-GFP-TamyB	Amp	Fig. 2, Supplementary	Chro6_2 neutral locus targeting plasmid

			Tables 4-5, Supplementary Figure 7	
JBx_236230	chro7_1:: pTEF1-GFP-TamyB	Amp	Fig. 2, Supplementary Tables 4-5, Supplementary Figure 7	Chro7_1 neutral locus targeting plasmid
JBx_236271	chro1-2:: An008cp-Bm3R1_VP16-TamyB	Amp	Fig. 3, Supplementary Table 6	Expression of the Bm3R1-VP16 sTF
JBx_236212	chro2-2::6xUAS-An_201205-mCherry-TamyB	Amp	Fig. 3, Supplementary Table 6	Evaluation of core promoter in SES
JBx_250930	chro2-2::An_201205-mCherry-TamyB	Amp	Fig. 3, Supplementary Table 6	Control containing no UAS
JBx_236213	chro2-2::6xUAS-Ao_aspnd1-mCherry-TamyB	Amp	Fig. 3, Supplementary Table 6	Evaluation of core promoter in SES
JBx_236214	chro2-2::6xUAS-Ao_amy1-mCherry-TamyB	Amp	Fig. 3, Supplementary Table 6	Evaluation of core promoter in SES
JBx_236215	chro2-2::6xUAS-Ao_gpdA-mCherry-TamyB	Amp	Fig. 3, Supplementary Table 6	Evaluation of core promoter in SES
JBx_236216	chro2-2::6xUAS-Ao_0666-mCherry-TamyB	Amp	Fig. 3, Supplementary Table 6	Evaluation of core promoter in SES
JBx_236217	chro2-2::6xUAS-Ao_prxA-mCherry-TamyB	Amp	Fig. 3, Supplementary Table 6	Evaluation of core promoter in SES
JBx_236218	chro2-2::6xUAS-Ao_0336-mCherry-TamyB	Amp	Fig. 3, Supplementary Table 6	Evaluation of core promoter in SES
JBx_236219	chro2-2::6xUAS-Ao_0583-mCherry-TamyB	Amp	Fig. 3, Supplementary Table 6	Evaluation of core promoter in SES
JBx_236220	chro2-2::6xUAS-Ao_pdcA-mCherry-TamyB	Amp	Fig. 3, Supplementary Table 6	Evaluation of core promoter in SES
JBx_236221	chro2-2::6xUAS-Ao_hhfA-mCherry-TamyB	Amp	Fig. 3, Supplementary Table 6	Evaluation of core promoter in SES
JBx_236222	chro2-2::6xUAS-pAmyB-mCherry-TamyB	Amp	Fig. 3, Supplementary Table 6	Evaluation of core promoter in SES
JBx_236223	chro2-2::6xUAS-pTEF1-mCherry-TamyB	Amp	Fig. 3, Supplementary Table 6	Evaluation of core promoter in SES
JBx_236224	chro1-3:: TtrpC-GFP-Ao_hhf-2xUAS-Ao_gpdA-GFP-TamyB	Amp	Fig. 3, Supplementary Table 6	Synthetic bidirectional promoter

JBx_250932	chro1-3:: TtrpC-GFP-p2_1-GFP-TamyB	Amp	Fig,3, Supplementary Figure 11, Supplementary Table 7	Endogenous bidirectional promoter
JBx_250934	chro1-3:: TtrpC-GFP-p4_2-GFP-TamyB	Amp	Fig,3, Supplementary Figure 11, Supplementary Table 7	Endogenous bidirectional promoter
JBx_250936	chro1-3::pTEF1-Egt1-TamyB	Amp	Fig. 4, Supplementary Figure 12	Ergothioneine engineering
JBx_250938	chro2-2::pTEF1-Egt2-TamyB	Amp	Fig. 4, Supplementary Figure 12	Ergothioneine engineering
JBx_250940	chro1-3:: TtrpC-Egt1-p4_2-Egt2-TamyB	Amp	Fig. 4, Supplementary Figure 12	Ergothioneine engineering
JBx_250942	chro1-3::pTEF1-LegH-TamyB	Amp	Fig. 4, Supplementary Figure 15	Heme engineering
JBx_250944	chro1-2::pAmyB-ALASdHRM-TamyB	Amp	Fig. 4, Supplementary Figure 15	Heme engineering-ALAS HRM mutant
JBx_250946	chro5-1::pAmyB-hemC-TamyB	Amp	Fig. 4, Supplementary Figure 15	Heme engineering
JBx_250948	chro2-2::pTEF1-UROD-TamyB	Amp	Fig. 4, Supplementary Figure 15	Heme engineering
JBx_250950	chro7-1::pTEF1-CPO-TamyB	Amp	Fig. 4, Supplementary Figure 15	Heme engineering
JBx_236225	chro6-2::An008cp-Bm3R1_VP16-TtrpC-6xUAS-AO0583cp-LegH-TamyB	Amp	Fig. 4, Supplementary Figure 15	Heme engineering

Supplementary Table 12. Wild-type and genetically modified *A. oryzae* strains used in this study.

Strain	Parent strain	Description	Auxotrophy	Comment	Reference
RIB40	N/A	wild-type *A. oryzae* RIB40	None		ATCC
RIB40 *pyrG*	RIB40	*A. oryzae* RIB40 *pyrG* mutant	*pyrG*	Uracil-uridine auxotroph	13
NRRL 1911	N/A	wild-type *A. oryzae* NRRL 1911	None		NRRL
NRRL 32614	N/A	wild-type *A. oryzae* NRRL 32614	None		NRRL
NRRL 5592	N/A	wild-type *A. oryzae* NRRL 5592	None		NRRL
NRRL 2218	N/A	wild-type *A. oryzae* NRRL 2218	None		NRRL
NRRL 6574	N/A	wild-type *A. oryzae* NRRL 6574	None		NRRL
JBx_250952	N/A	*A. oryzae* NRRL 1911 *pyrG mutant*	*pyrG*	Uracil-uridine auxotroph	This study
JBx_250953	N/A	*A. oryzae* NRRL 32614 *pyrG mutant*	*pyrG*	Uracil-uridine auxotroph	This study
JBx_250954	N/A	*A. oryzae* NRRL 5592 *pyrG mutant*	*pyrG*	Uracil-uridine auxotroph	This study
JBx_250955	N/A	*A. oryzae* NRRL 2218 *pyrG mutant*	*pyrG*	Uracil-uridine auxotroph	This study
JBx_250956	N/A	*A. oryzae* NRRL 6574 *pyrG mutant*	*pyrG*	Uracil-uridine auxotroph	This study
JBx_236201	RIB40	chro1-2::An008cp-Bm3R1_VP16-TamyB	*pyrG*	Strain expressing the Bm3R1-VP16 sTF	This study
JBx_250957	RIB40	chro1-3::pTEF1-Egt1-TamyB chro2-2::pTEF1-Egt2-TamyB	None	Strain engineered for ergothioneine overproduction (VMR_Eg1_2) Expressing AO_Egt1 and AO_Egt2 from two separate promoters	This study

JBx_250958	RIB40	chro1-3:: TtrpC-Egt1-p4_2-Egt2-TamyB	None	Strain engineered for ergothioneine overproduction (VMR_Eg1-2)<br>Expressing AO_Egt1 and AO_Egt2 from the bidirectional promoter p4_2	This study
JBx_250959	RIB40	chro1-3::pTEF1-LegH-TamyB<br>chro1-2::pAmyB-ALASdHRM-TamyB<br>chro5-1::pAmyB-hemC-TamyB<br>chro2-2::pTEF1-UROD-TamyB<br>chro7-1::pTEF1-CPO-TamyB<br>chro6-2::An008cp-Bm3R1_VP16-TtrpC-6xUAS-AO0583cp-LegH-TamyB	None	Strain engineered for increased heme production (VMR-HEM_v1)	This study

Supplementary Table 13. Dataset used for transcriptome mining for candidate neutral integration sites and bidirectional promoters in *A. oryzae*.

Set	Run	BioSample	Bases	Experiment	Time point
1	DRR178650	SAMD00171570	4.81 G	DRX169176	48 h
2	DRR178651	SAMD00171571	4.76 G	DRX169177	48 h
3	DRR178652	SAMD00171572	4.42 G	DRX169178	48 h
4	DRR178653	SAMD00171573	4.47 G	DRX169179	72 h
5	DRR178654	SAMD00171574	4.17 G	DRX169180	72 h
6	DRR178655	SAMD00171575	4.27 G	DRX169181	72 h
7	DRR178656	SAMD00171576	4.40 G	DRX169182	96 h
8	DRR178657	SAMD00171577	4.45 G	DRX169183	96 h
9	DRR178658	SAMD00171578	4.80 G	DRX169184	96 h
10	DRR178659	SAMD00171579	4.75 G	DRX169185	120 h
11	DRR178660	SAMD00171580	4.70 G	DRX169186	120 h
12	DRR178661	SAMD00171581	4.50 G	DRX169187	120 h
13	DRR178662	SAMD00171582	4.45 G	DRX169188	144 h
14	DRR178663	SAMD00171583	4.49 G	DRX169189	144 h
15	DRR178664	SAMD00171584	4.49 G	DRX169190	144 h
16	DRR178665	SAMD00171585	4.44 G	DRX169191	168 h
17	DRR178666	SAMD00171586	4.53 G	DRX169192	168 h
18	DRR178667	SAMD00171587	4.26 G	DRX169193	168 h

References

1 Stone, W. E. & Wright, H. E. NOTES ON TAKA-DIASTASE. *Journal of the American Chemical Society* **20**, 639-647 (1898). https://doi.org:10.1021/ja02071a001

2 Katayama, T. *et al.* Forced Recycling of an AMA1-Based Genome-Editing Plasmid Allows for Efficient Multiple Gene Deletion/Integration in the Industrial Filamentous Fungus Aspergillus oryzae. *Appl Environ Microbiol* **85** (2019). https://doi.org:10.1128/AEM.01896-18

3 Yoon, J., Kikuma, T., Maruyama, J. & Kitamoto, K. Enhanced production of bovine chymosin by autophagy deficiency in the filamentous fungus Aspergillus oryzae. *PLoS One* **8**, e62512 (2013). https://doi.org:10.1371/journal.pone.0062512

4 Rantasalo, A. *et al.* A universal gene expression system for fungi. *Nucleic Acids Res* **46**, e111 (2018). https://doi.org:10.1093/nar/gky558

5 Umemura, M., Kuriiwa, K., Dao, L. V., Okuda, T. & Terai, G. Promoter tools for further development of Aspergillus oryzae as a platform for fungal secondary metabolite production. *Fungal Biol Biotechnol* **7**, 3 (2020). https://doi.org:10.1186/s40694-020-00093-1

6 Hu, W. *et al.* Bioinformatic and biochemical characterizations of C-S bond formation and cleavage enzymes in the fungus Neurospora crassa ergothioneine biosynthetic pathway. *Org Lett* **16**, 5382-5385 (2014). https://doi.org:10.1021/ol502596z

7 Irani, S. *et al.* Snapshots of C-S Cleavage in Egt2 Reveals Substrate Specificity and Reaction Mechanism. *Cell Chem Biol* **25**, 519-529 e514 (2018). https://doi.org:10.1016/j.chembiol.2018.02.002

8 Wang, L. Z. *et al.* Quantification of L-ergothioneine in human plasma and erythrocytes by liquid chromatography-tandem mass spectrometry. *J Mass Spectrom* **48**, 406-412 (2013). https://doi.org:10.1002/jms.3150

9 Liu, L., Martinez, J. L., Liu, Z., Petranovic, D. & Nielsen, J. Balanced globin protein expression and heme biosynthesis improve production of human hemoglobin in Saccharomyces cerevisiae. *Metab Eng* **21**, 9-16 (2014). https://doi.org:10.1016/j.ymben.2013.10.010

10 Zoładek, T., Nguyen, B. N. & Rytka, J. Saccharomyces cerevisiae mutants defective in heme biosynthesis as a tool for studying the mechanism of phototoxicity of porphyrins. *Photochem Photobiol* **64**, 957-962 (1996). https://doi.org:10.1111/j.1751-1097.1996.tb01861.x

11 Michener, J. K., Nielsen, J. & Smolke, C. D. Identification and treatment of heme depletion attributed to overexpression of a lineage of evolved P450 monooxygenases. *Proc Natl Acad Sci U S A* **109**, 19504-19509 (2012). https://doi.org:10.1073/pnas.1212287109

12 Sana, T. R., Waddell, K. & Fischer, S. M. A sample extraction and chromatographic strategy for increasing LC/MS detection coverage of the erythrocyte metabolome. *J Chromatogr B Analyt Technol Biomed Life Sci* **871**, 314-321 (2008). https://doi.org:10.1016/j.jchromb.2008.04.030

13 Okabe, T. *et al.* BiFC-based visualisation system reveals cell fusion morphology and heterokaryon incompatibility in the filamentous fungus Aspergillus oryzae. *Sci Rep* **8**, 2922 (2018). https://doi.org:10.1038/s41598-018-21323-y
