# Supplementary material for: Edible mycelium bioengineered for enhanced nutritional value and sensory appeal using a modular synthetic biology toolkit
Source: Nat Commun. 2024 Mar 14;15:2099. doi: 10.1038/s41467-024-46314-8 (PMC10940619; doi:10.1038/s41467-024-46314-8)
Supplement: Supplementary file 3 — Description of Additional Supplementary Files [file 41467_2024_46314_MOESM3_ESM.pdf]

Description of Additional Supplementary Files

Supplementary Data 1. Computationally identified potential neutral loci for high gene expression. Candidate neutral loci for integration. These loci are flanked by highly expressed genes found in 18 conditions. The genes were identified by read count-based ranking. The details of how these sequences were selected can be found in the "Computational identification of candidate neutral, highly transcribed integration sites for protein expression" section of the supplementary file associated with this manuscript. The data are filtered by length >4499 bp and >15 occurrences. A total of 10 loci were tested in this study. Those are shown in Table S3.

Supplementary Data 2. Candidate endogenous A. oryzae bidirectional promoters identified from available transcriptomics data in A. oryzae. 5' and 3' base indicate the starting base of the genes pointing in opposite directions; the candidate bidirectional promoter is in between these bases in the genome. Frequency is the percentage of datasets where this was a highly ranked region across the 18 transcriptomics datasets that were analyzed. Five total candidate bidirectional promoters were analyzed.
