# Supplementary material for: Edible mycelium bioengineered for enhanced nutritional value and sensory appeal using a modular synthetic biology toolkit
Source: Nat Commun. 2024 Mar 14;15:2099. doi: 10.1038/s41467-024-46314-8 (PMC10940619; doi:10.1038/s41467-024-46314-8)
Supplement: Supplementary file 6 — Reporting Summary [file 41467_2024_46314_MOESM6_ESM.pdf]

nature portfolio

Corresponding author(s): Jay D. Keasling

Last updated by author(s): Feb 2, 2024

Reporting Summary

Nature Portfolio wishes to improve the reproducibility of the work that we publish. This form provides structure for consistency and transparency in reporting. For further information on Nature Portfolio policies, see our Editorial Policies and the Editorial Policy Checklist.

Statistics

For all statistical analyses, confirm that the following items are present in the figure legend, table legend, main text, or Methods section.

n/a	Confirmed	
☐	☒	The exact sample size (*n*) for each experimental group/condition, given as a discrete number and unit of measurement
☐	☒	A statement on whether measurements were taken from distinct samples or whether the same sample was measured repeatedly
☐	☒	The statistical test(s) used AND whether they are one- or two-sided<br>*Only common tests should be described solely by name; describe more complex techniques in the Methods section.*
☒	☐	A description of all covariates tested
☒	☐	A description of any assumptions or corrections, such as tests of normality and adjustment for multiple comparisons
☐	☒	A full description of the statistical parameters including central tendency (e.g. means) or other basic estimates (e.g. regression coefficient) AND variation (e.g. standard deviation) or associated estimates of uncertainty (e.g. confidence intervals)
☐	☒	For null hypothesis testing, the test statistic (e.g. *F*, *t*, *r*) with confidence intervals, effect sizes, degrees of freedom and *P* value noted<br>*Give P values as exact values whenever suitable.*
☒	☐	For Bayesian analysis, information on the choice of priors and Markov chain Monte Carlo settings
☒	☐	For hierarchical and complex designs, identification of the appropriate level for tests and full reporting of outcomes
☒	☐	Estimates of effect sizes (e.g. Cohen's *d*, Pearson's *r*), indicating how they were calculated

Our web collection on statistics for biologists contains articles on many of the points above.

Software and code

Policy information about availability of computer code

Data collection	The bioinformatics methods used in the study are standard and based on existing software that was developed by others and already published in the literature. The software has been properly cited in the methods. The relevant command lines have been placed in the supplementary material and the instructions for reproducing these experiments have been clearly stated in the supplementary materials.
Data analysis	No custom software was used. The software has been properly cited in the methods and the data availability of the manuscript, as described above. Analysis and assembly of A. oryzae genomes and analysis of their biosynthetic gene clusters was performed using a combination of TrimmomaticPE version 0.39, SPAdes genome assembler v3.13.1-1, AUGUSTUS v3.4.0, and antiSMASH version 7. Phylogenetic analysis of core genomes was conducted using IQtree2 v2.0.7, in a process executed automatically using a script available at https://github.com/WeMakeMolecules/Core-to-Tree. To align sequences to the A. oryzae reference genome for identification of highly expressed neutral loci, subread package v 2.0.3 was used. Highly expressed genes were identified using featureCounts v2.0.3. All sequence alignments were performed using Snapgene (v.7.1.1). Microscopy images were captured and processed using the Leica LAS X software (v.5.1.0). Statistical analysis of expression levels was performed using Graphpad Prism (v.10.1.1). FlowJo software version 10 was used for analyzing and quantifying data from flow cytometry experiments. BioRender (http://BioRender.com) was used in the construction of specific figures (this information is included in the caption if relevant).

For manuscripts utilizing custom algorithms or software that are central to the research but not yet described in published literature, software must be made available to editors and reviewers. We strongly encourage code deposition in a community repository (e.g. GitHub). See the Nature Portfolio guidelines for submitting code & software for further information.

Data

Policy information about availability of data

All manuscripts must include a data availability statement. This statement should provide the following information, where applicable:

- Accession codes, unique identifiers, or web links for publicly available datasets
- A description of any restrictions on data availability
- For clinical datasets or third party data, please ensure that the statement adheres to our policy

The genome sequences of the A. oryzae strains obtained from NRRL and sequenced as part of this study have been deposited to the Sequence Read Archive under Bioproject #PRJNA987873. The generated mass spectrometry proteomics data have been deposited to the ProteomeXchange Consortium via the PRIDE partner repository with the dataset identifier PXD043152. DIA-NN is freely available for download from https://github.com/vdemichev/DiaNN.
The transcriptome data used to identify endogenous neutral loci and bidirectional promoters in Aspergillus oryzae can be found in GenBank under BioProject accession: PRJDB8293. CAoGD (Comprehensive Aspergillus oryzae Genome Database) v.2.4 was used to identify the genomic location and sequence identity of endogenous promoters and genes targeted in transformations. Data generated as part of the study will be made available upon request.

Research involving human participants, their data, or biological material

Policy information about studies with human participants or human data. See also policy information about sex, gender (identity/presentation), and sexual orientation and race, ethnicity and racism.

Reporting on sex and gender	N/A
Reporting on race, ethnicity, or other socially relevant groupings	N/A
Population characteristics	N/A
Recruitment	N/A
Ethics oversight	N/A

Note that full information on the approval of the study protocol must also be provided in the manuscript.

Field-specific reporting

Please select the one below that is the best fit for your research. If you are not sure, read the appropriate sections before making your selection.

☒ Life sciences ☐ Behavioural & social sciences ☐ Ecological, evolutionary & environmental sciences

For a reference copy of the document with all sections, see nature.com/documents/nr-reporting-summary-flat.pdf

Life sciences study design

All studies must disclose on these points even when the disclosure is negative.

Sample size	n=3 was chosen as the minimal number of replicates for experimental characterization. We determined this to be sufficient based on internal controls (using previously characterized promoters and fluorescent genes) to capture biological variability between transformants/strains.
Data exclusions	No data were excluded
Replication	Experiments were performed at least twice independently, unless otherwise noted in the text
Randomization	All transformed colonies were selected at random for PCR-based screening. A subset of colonies that were confirmed by PCR as harboring the insertion of interest were analyzed equally, with no additional sub-sampling. Therefore there was no further requirement for randomization.
Blinding	Blinding was not applicable as experimental conditions were evident from the experimental procedure (eg. engineered/nonengineered).

Reporting for specific materials, systems and methods

We require information from authors about some types of materials, experimental systems and methods used in many studies. Here, indicate whether each material, system or method listed is relevant to your study. If you are not sure if a list item applies to your research, read the appropriate section before selecting a response.

Materials & experimental systems

n/a	Involved in the study
☒	☐ Antibodies
☒	☐ Eukaryotic cell lines
☒	☐ Palaeontology and archaeology
☒	☐ Animals and other organisms
☒	☐ Clinical data
☒	☐ Dual use research of concern
☒	☐ Plants

Methods

n/a	Involved in the study
☒	☐ ChIP-seq
☐	☒ Flow cytometry
☒	☐ MRI-based neuroimaging

Flow Cytometry

Plots

Confirm that:

☒ The axis labels state the marker and fluorochrome used (e.g. CD4-FITC).

☐ The axis scales are clearly visible. Include numbers along axes only for bottom left plot of group (a 'group' is an analysis of identical markers).

☐ All plots are contour plots with outliers or pseudocolor plots.

☐ A numerical value for number of cells or percentage (with statistics) is provided.

Methodology

Sample preparation	Conidia of A. oryzae constutively expression fluorescent proteins were obtained as a suspension from slants after 5-7 days of growth, as detailed in the methods.
Instrument	BD Accuri C6
Software	The provided BD Accuri C6 data collection software was used for data collection. FlowJo software version 10 was used for analyzing and quantifying data (mean fluorescence intensity across the population, in each sample).
Cell population abundance	No sorting was performed; the mean fluorescence intensity was measured on the bulk population for all collected events (50,000 events).
Gating strategy	No gating strategy was performed. The the FSC-H threshold was set as >80,000 for during data collection, based on the manufacturer's recommendations and literature precedent.

☐ Tick this box to confirm that a figure exemplifying the gating strategy is provided in the Supplementary Information.
