# Supplementary material for: Edible mycelium bioengineered for enhanced nutritional value and sensory appeal using a modular synthetic biology toolkit
Source: Nat Commun. 2024 Mar 14;15:2099. doi: 10.1038/s41467-024-46314-8 (PMC10940619; doi:10.1038/s41467-024-46314-8)
Supplement: Supplementary file 7 — Source data [file 41467_2024_46314_MOESM7_ESM.zip › Source data /Mass spectrometry/Supplementary Figure 16_masspec_output.pptx]

## Slide 1
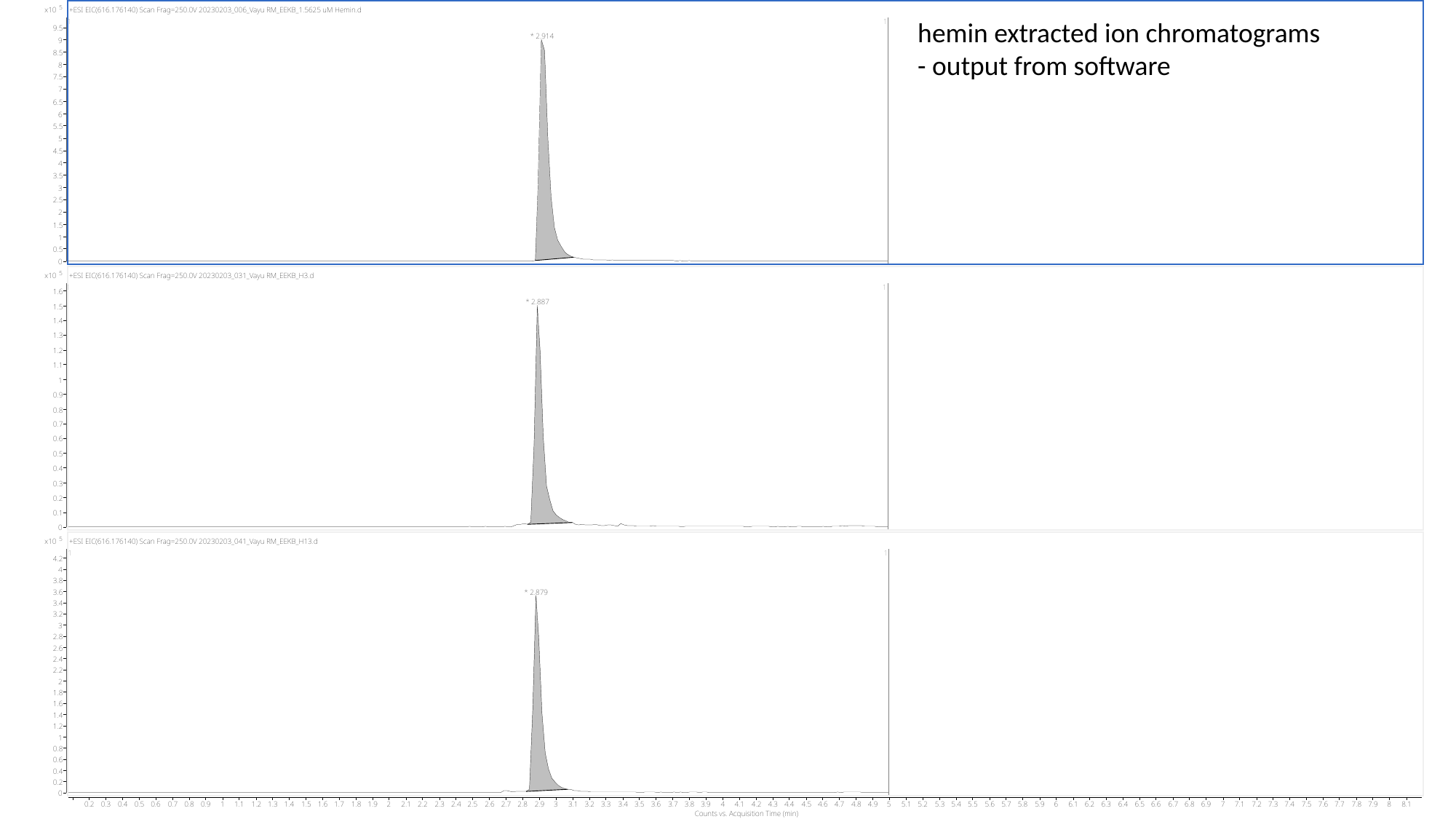

hemin extracted ion chromatograms
- output from software

## Slide 2
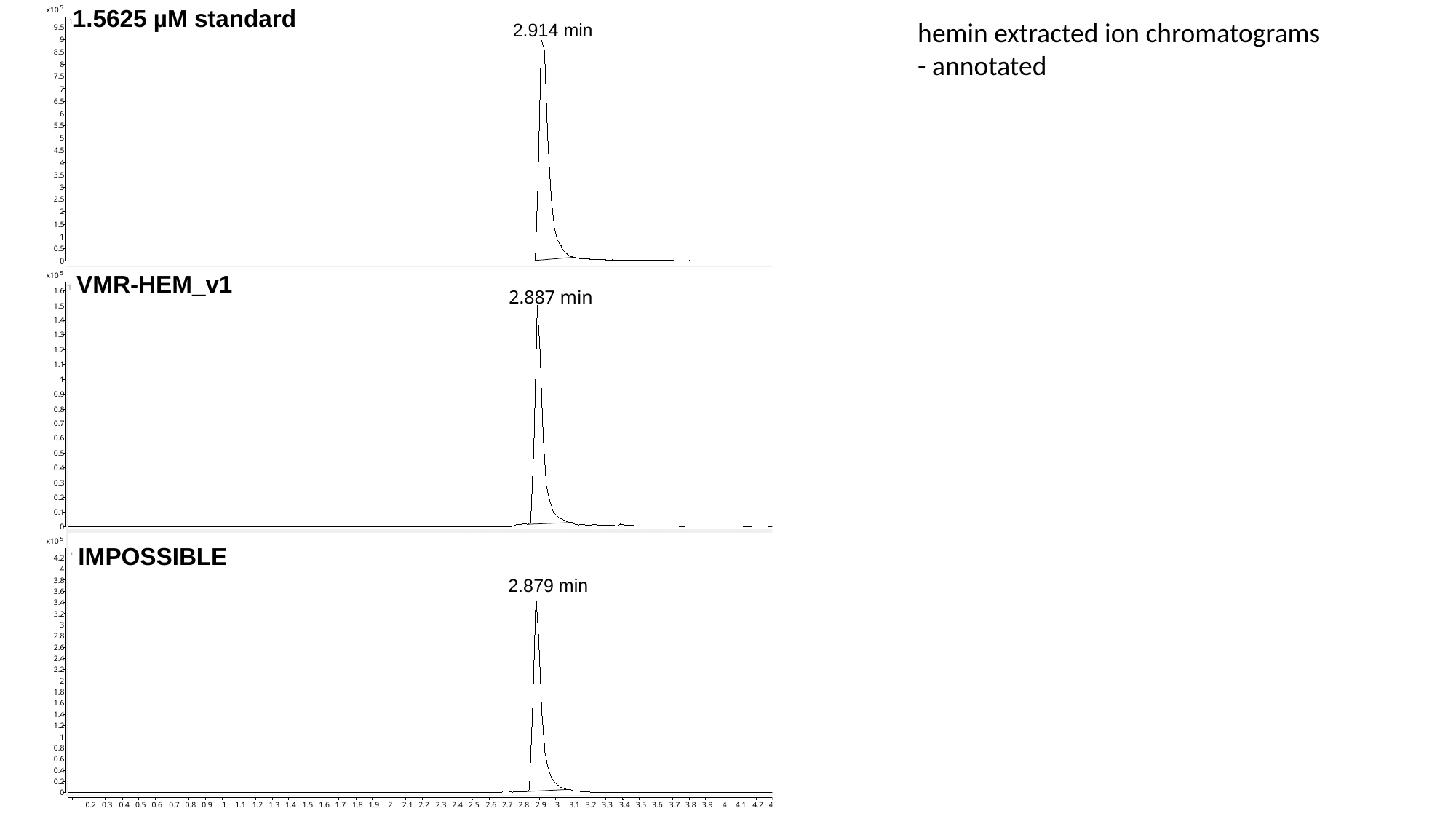

5
+ESI EIC(616.176140) Scan Frag=250.0V 20230203_006_Vayu RM_EEKB_1.5625 uM Hemin.d
x10
1
 2.914 min
Hemin extracted ion chromatograms
9.5
9
8.5
8
7.5
7
6.5
6
5.5
5
4.5
4
3.5
3
2.5
2
1.5
1
0.5
0
5
+ESI EIC(616.176140) Scan Frag=250.0V 20230203_031_Vayu RM_EEKB_H3.d
x10
1
2.887 min
1.6
1.5
1.4
1.3
1.2
1.1
1
0.9
0.8
0.7
0.6
0.5
0.4
0.3
0.2
0.1
0
5
+ESI EIC(616.176140) Scan Frag=250.0V 20230203_041_Vayu RM_EEKB_H13.d
x10
1
4.2
4
2.879 min
3.8
3.6
3.4
3.2
3
2.8
2.6
2.4
2.2
2
1.8
1.6
1.4
1.2
1
0.8
0.6
0.4
0.2
0
0.2
0.3
0.4
0.5
0.6
0.7
0.8
0.9
1
1.1
1.2
1.3
1.4
1.5
1.6
1.7
1.8
1.9
2
2.1
2.2
2.3
2.4
2.5
2.6
2.7
2.8
2.9
3
3.1
3.2
3.3
3.4
3.5
3.6
3.7
3.8
3.9
4
4.1
4.2
4.3
4.4
4.5
4.6
4.7
4.8
4.9
5
5.1
5.2
5.3
5.4
5.5
5.6
5.7
5.8
5.9
6
6.1
6.2
6.3
6.4
6.5
6.6
6.7
6.8
6.9
7
7.1
7.2
7.3
7.4
7.5
7.6
7.7
7.8
7.9
8
8.1
1.5625 µM standard
hemin extracted ion chromatograms
- annotated
VMR-HEM_v1
IMPOSSIBLE
Counts vs. Acquisition Time (min)

## Slide 3
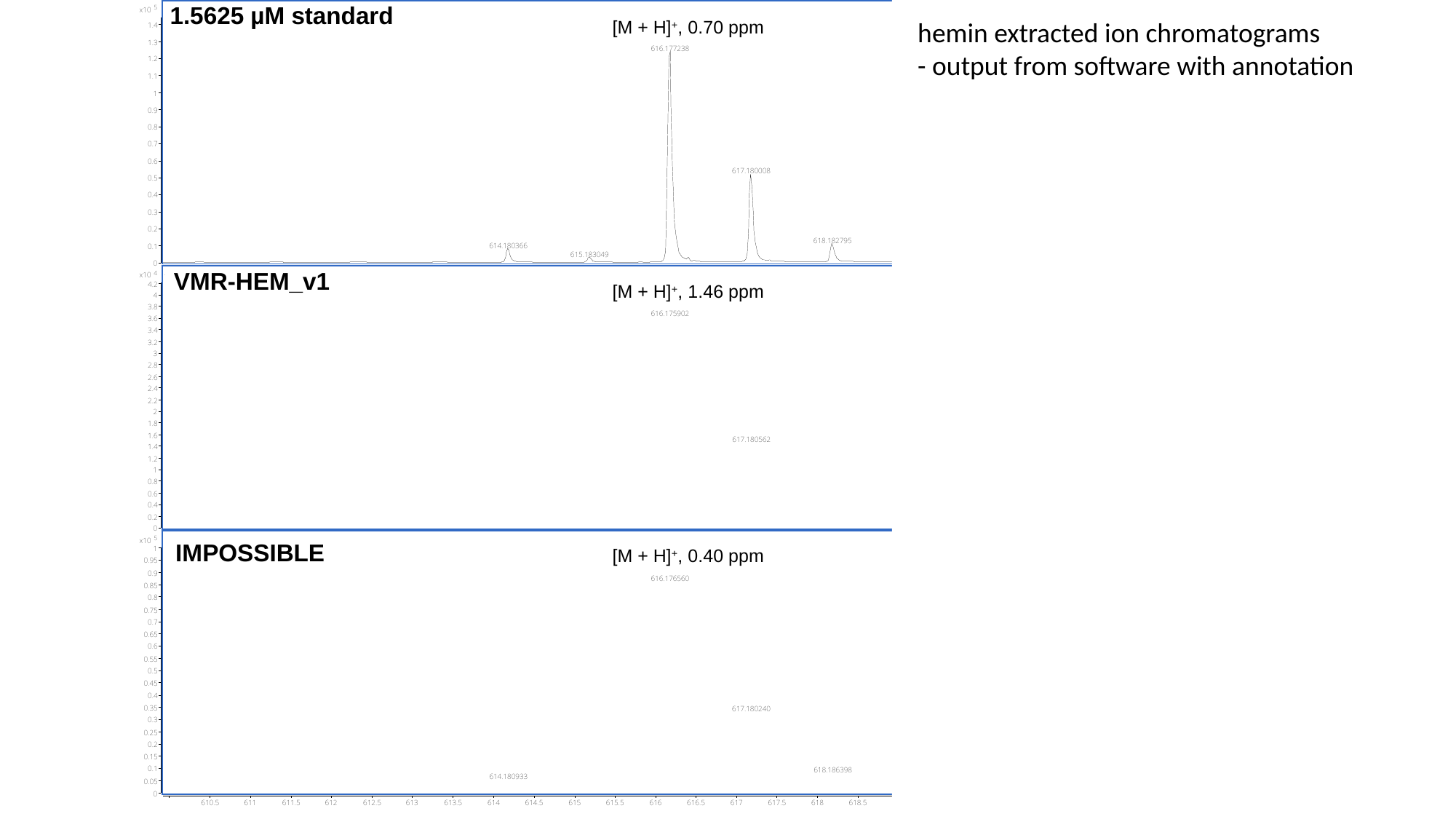

1.5625 µM standard
hemin extracted ion chromatograms
- output from software with annotation
[M + H]+, 0.70 ppm
VMR-HEM_v1
[M + H]+, 1.46 ppm
IMPOSSIBLE
[M + H]+, 0.40 ppm
Counts vs. Mass-to-Charge (m/z)

## Slide 4
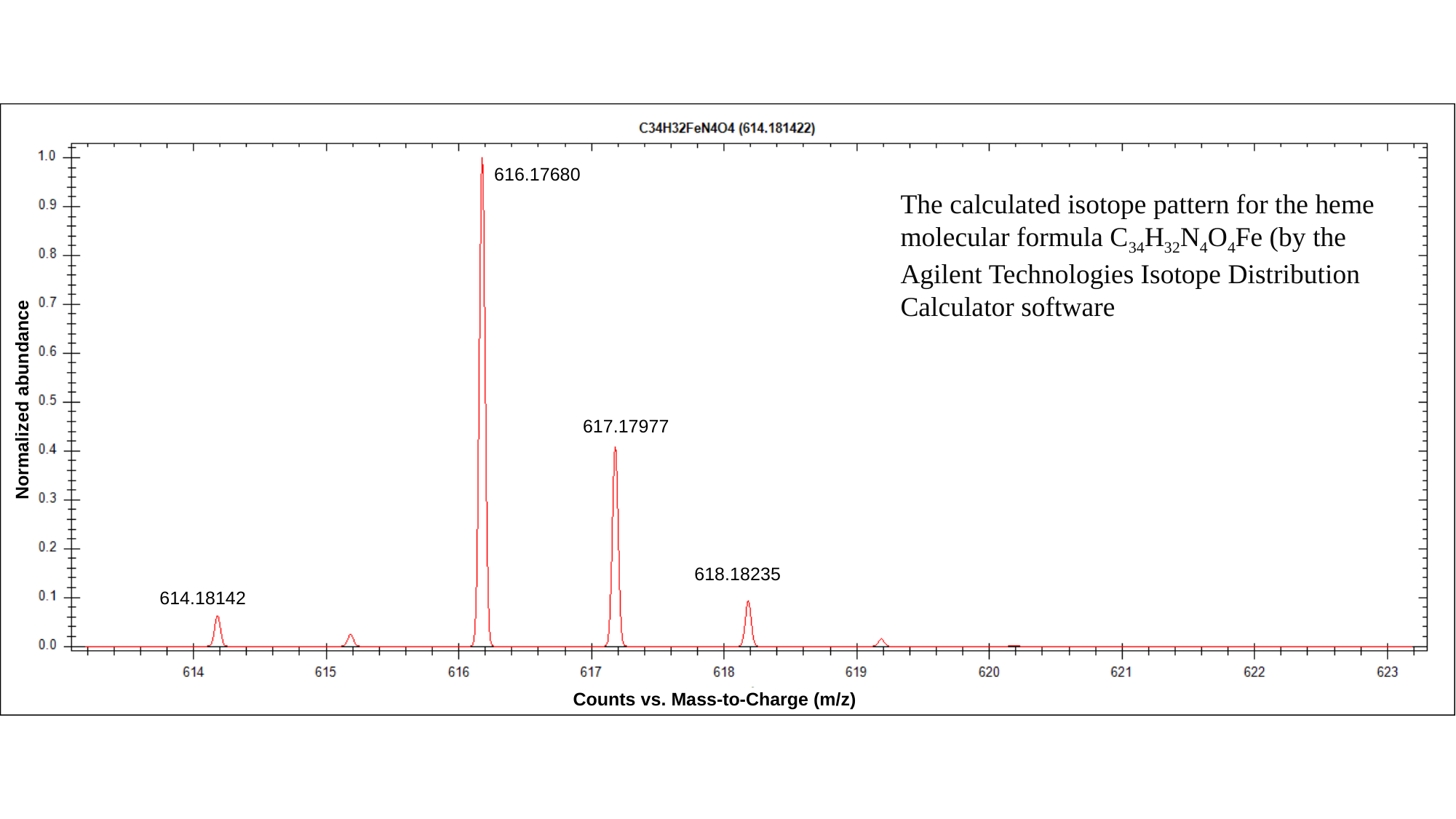

616.17680
The calculated isotope pattern for the heme molecular formula C34H32N4O4Fe (by the Agilent Technologies Isotope Distribution Calculator software
Normalized abundance
617.17977
618.18235
614.18142
Counts vs. Mass-to-Charge (m/z)
