# Supplementary material for: Edible mycelium bioengineered for enhanced nutritional value and sensory appeal using a modular synthetic biology toolkit
Source: Nat Commun. 2024 Mar 14;15:2099. doi: 10.1038/s41467-024-46314-8 (PMC10940619; doi:10.1038/s41467-024-46314-8)
Supplement: Supplementary file 7 — Source data [file 41467_2024_46314_MOESM7_ESM.zip › Source data /Mass spectrometry/Supplementary Figure 13_masspec_output.pptx]

## Slide 1
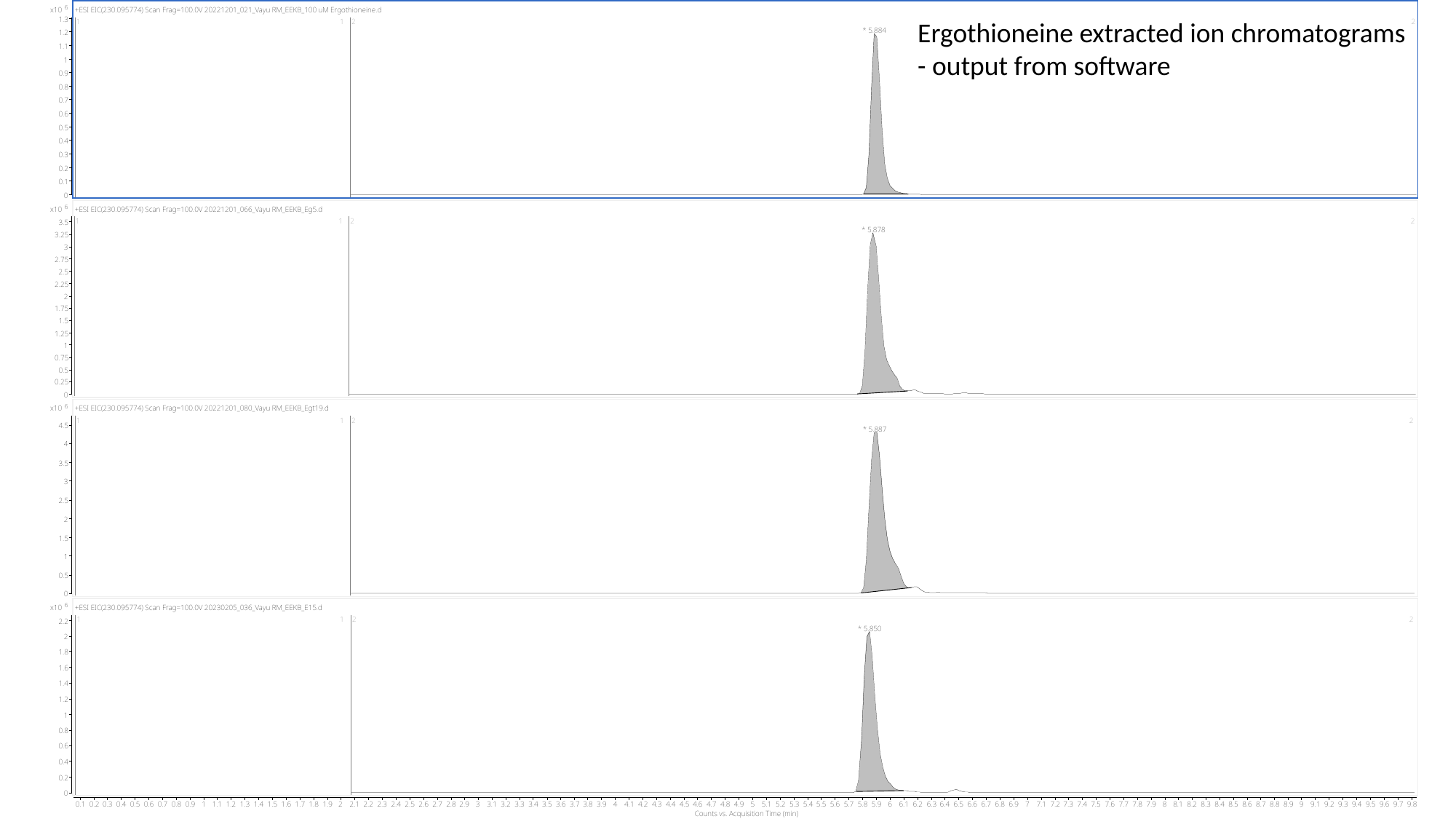

Ergothioneine extracted ion chromatograms
- output from software

## Slide 2
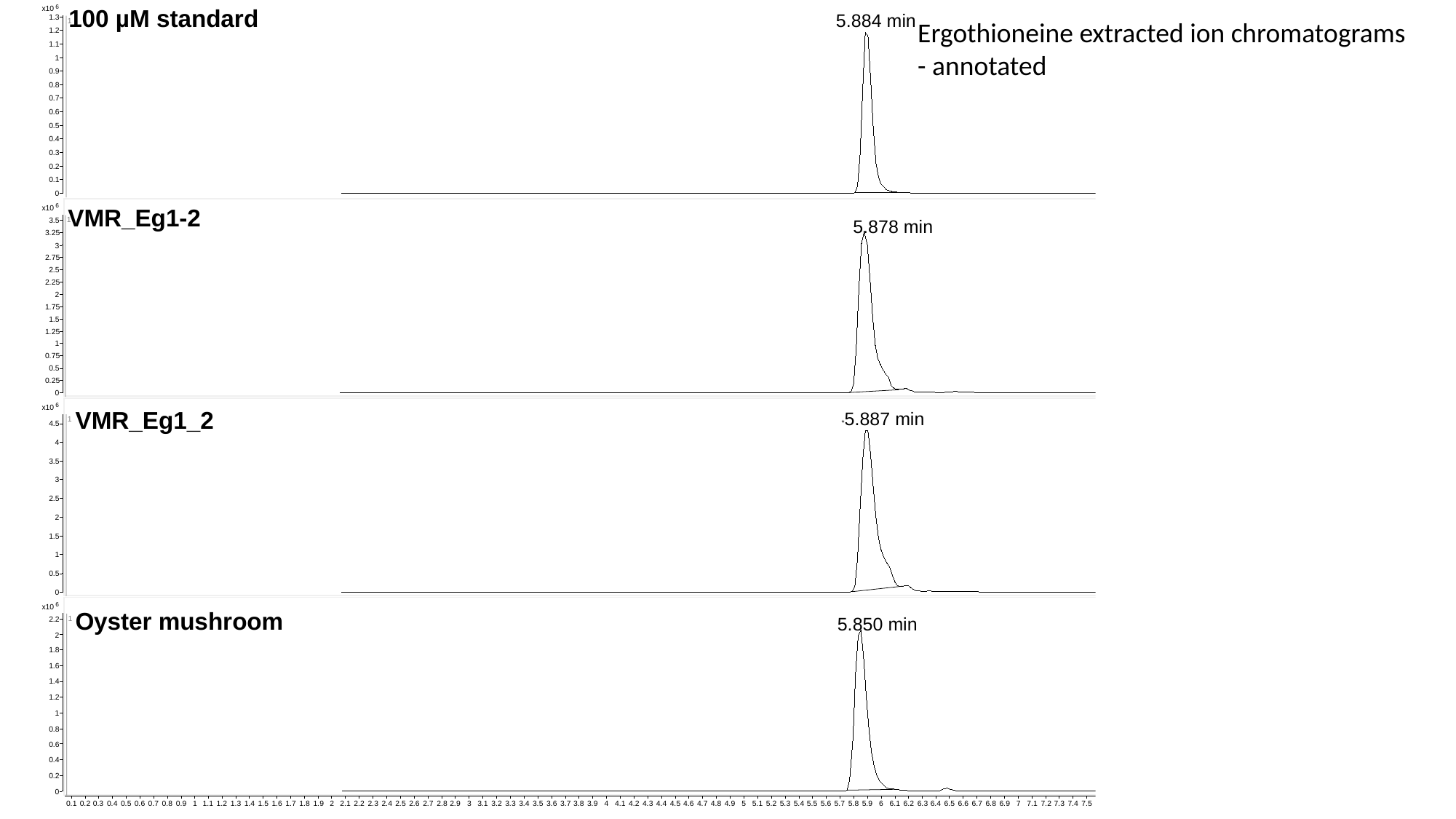

6
100 µM standard
x10
5.884 min
1.3
1
1.2
1.1
1
0.9
0.8
0.7
0.6
0.5
0.4
0.3
0.2
0.1
0
6
VMR_Eg1-2
x10
1
5.878 min
3.5
3.25
3
2.75
2.5
2.25
2
1.75
1.5
1.25
1
0.75
0.5
0.25
0
6
x10
*5.887 min
1
4.5
4
3.5
3
2.5
2
1.5
1
0.5
0
6
x10
5.850 min
1
2.2
2
1.8
1.6
1.4
1.2
1
0.8
0.6
0.4
0.2
0
0.1
0.2
0.3
0.4
0.5
0.6
0.7
0.8
0.9
1
1.1
1.2
1.3
1.4
1.5
1.6
1.7
1.8
1.9
2
2.1
2.2
2.3
2.4
2.5
2.6
2.7
2.8
2.9
3
3.1
3.2
3.3
3.4
3.5
3.6
3.7
3.8
3.9
4
4.1
4.2
4.3
4.4
4.5
4.6
4.7
4.8
4.9
5
5.1
5.2
5.3
5.4
5.5
5.6
5.7
5.8
5.9
6
6.1
6.2
6.3
6.4
6.5
6.6
6.7
6.8
6.9
7
7.1
7.2
7.3
7.4
7.5
7.6
7.7
7.8
7.9
8
8.1
8.2
8.3
8.4
8.5
8.6
8.7
8.8
8.9
9
9.1
9.2
9.3
9.4
9.5
9.6
9.7
9.8
Counts vs. Acquisition Time (min)
Ergothioneine extracted ion chromatograms
- annotated
VMR_Eg1_2
Oyster mushroom

## Slide 3
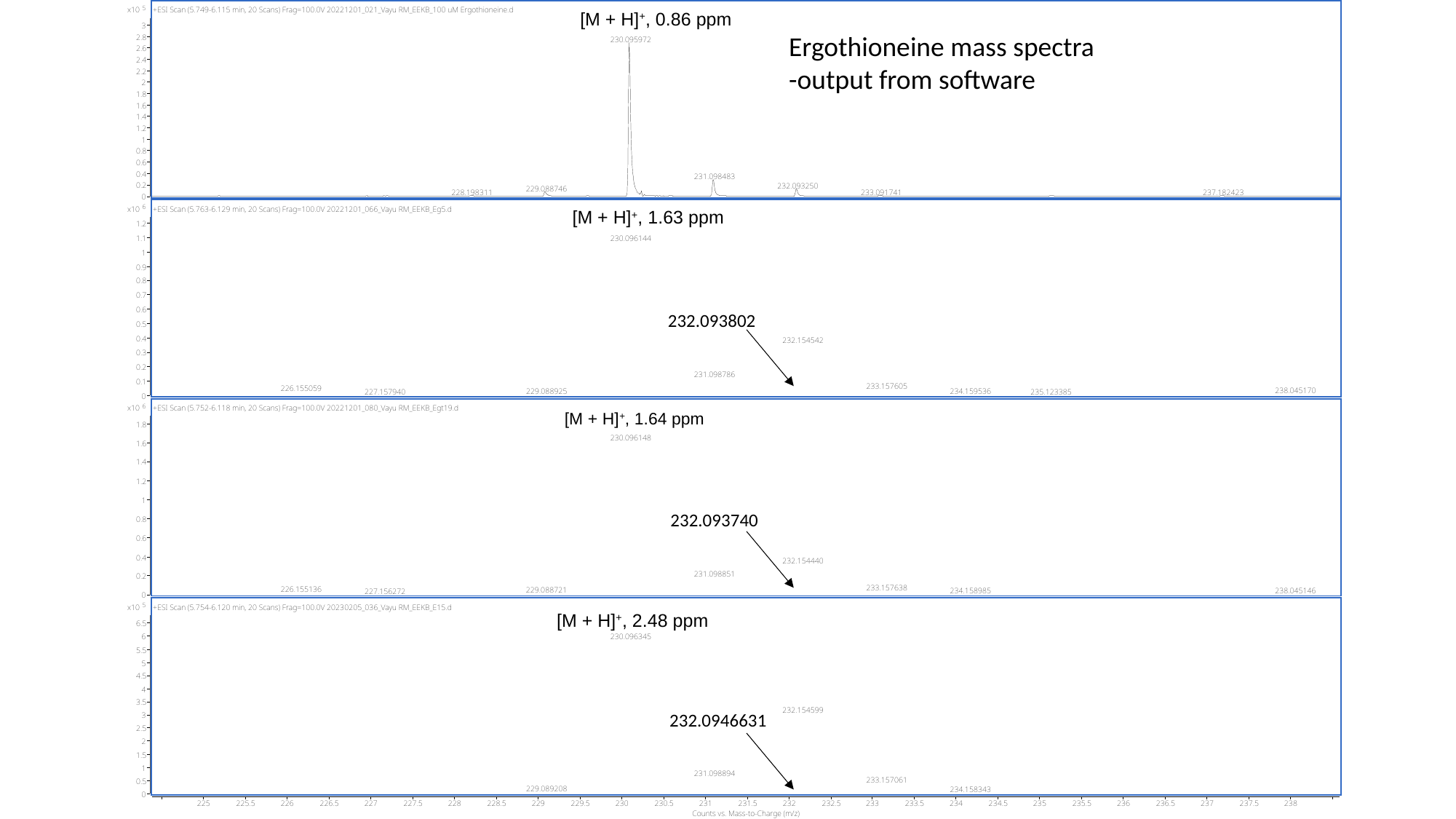

[M + H]+, 0.86 ppm
Ergothioneine mass spectra
-output from software
[M + H]+, 1.63 ppm
232.093802
[M + H]+, 1.64 ppm
232.093740
[M + H]+, 2.48 ppm
232.0946631

## Slide 4
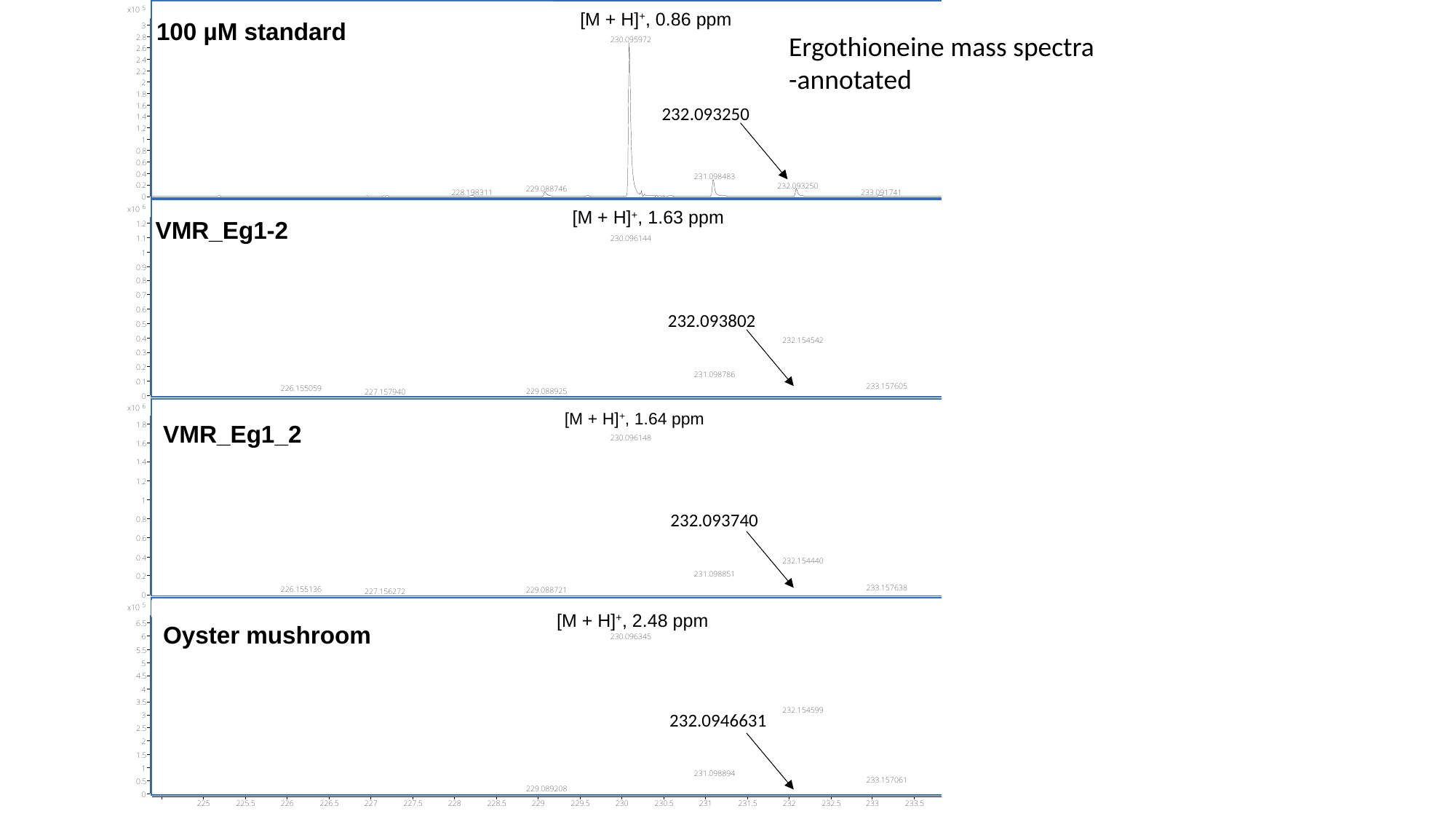

[M + H]+, 0.86 ppm
100 µM standard
Ergothioneine mass spectra
-annotated
232.093250
[M + H]+, 1.63 ppm
VMR_Eg1-2
232.093802
[M + H]+, 1.64 ppm
VMR_Eg1_2
232.093740
[M + H]+, 2.48 ppm
Oyster mushroom
232.0946631
Counts vs. Mass-to-Charge (m/z)
