# Supplementary figures and images for: Edible mycelium bioengineered for enhanced nutritional value and sensory appeal using a modular synthetic biology toolkit
Source: Nat Commun. 2024 Mar 14;15:2099. doi: 10.1038/s41467-024-46314-8 (PMC10940619; doi:10.1038/s41467-024-46314-8)

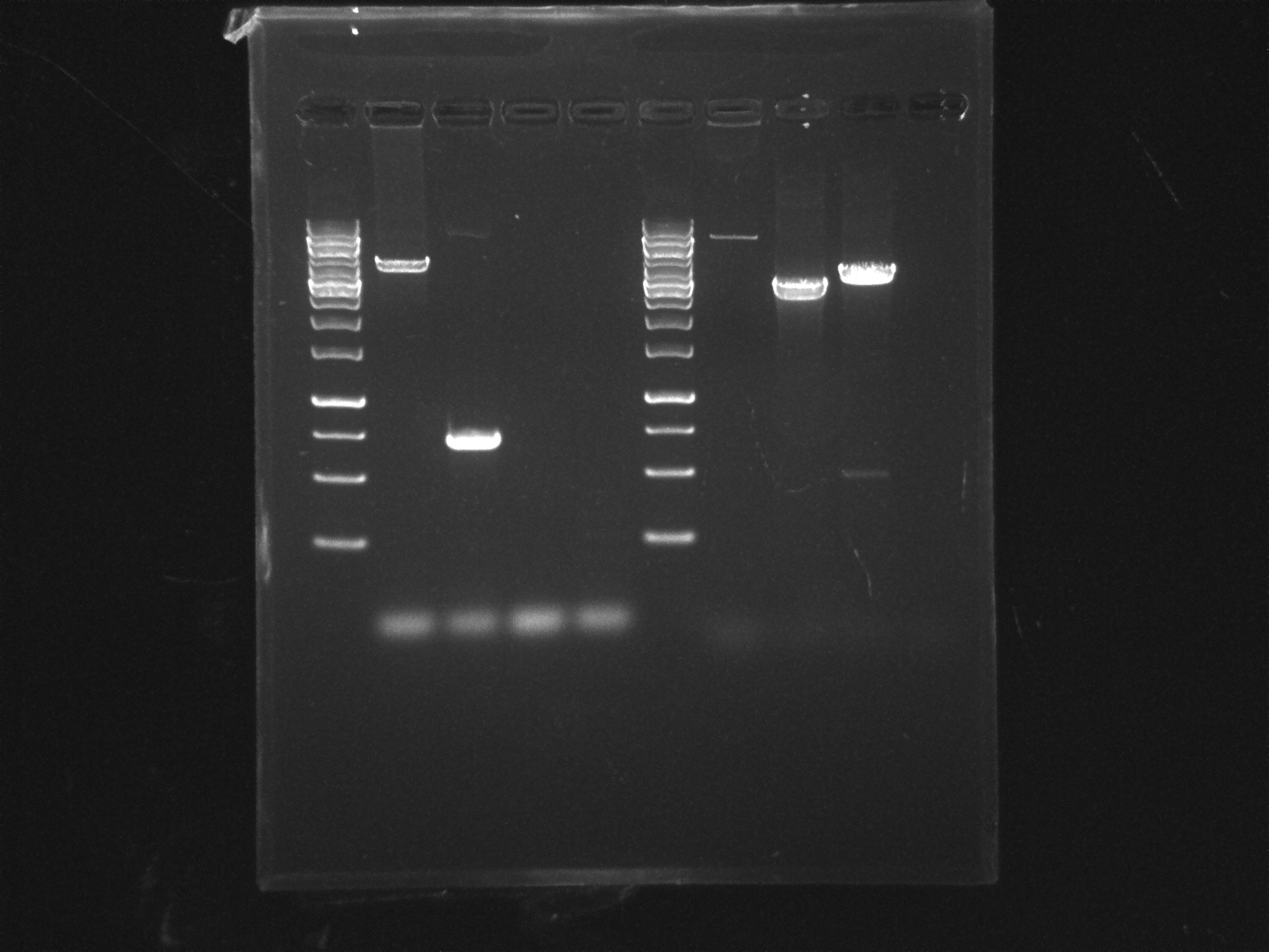

Supplement: Supplementary file 7 — Source data [file 41467_2024_46314_MOESM7_ESM.zip › Source data /Gels/Fig1G-gel.TIF]

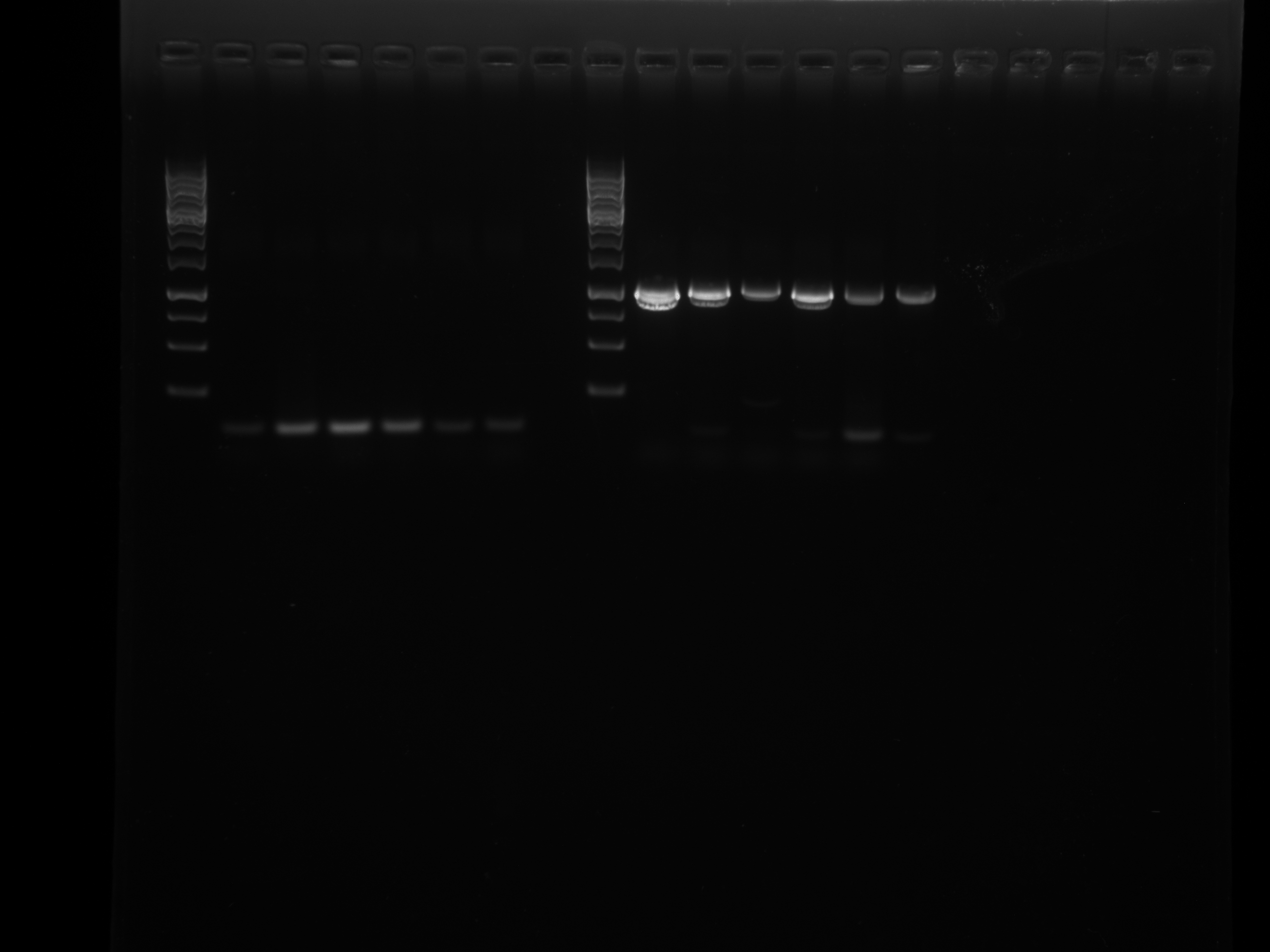

Supplement: Supplementary file 7 — Source data [file 41467_2024_46314_MOESM7_ESM.zip › Source data /Gels/Supplementary Figure 4D-gel.tif]

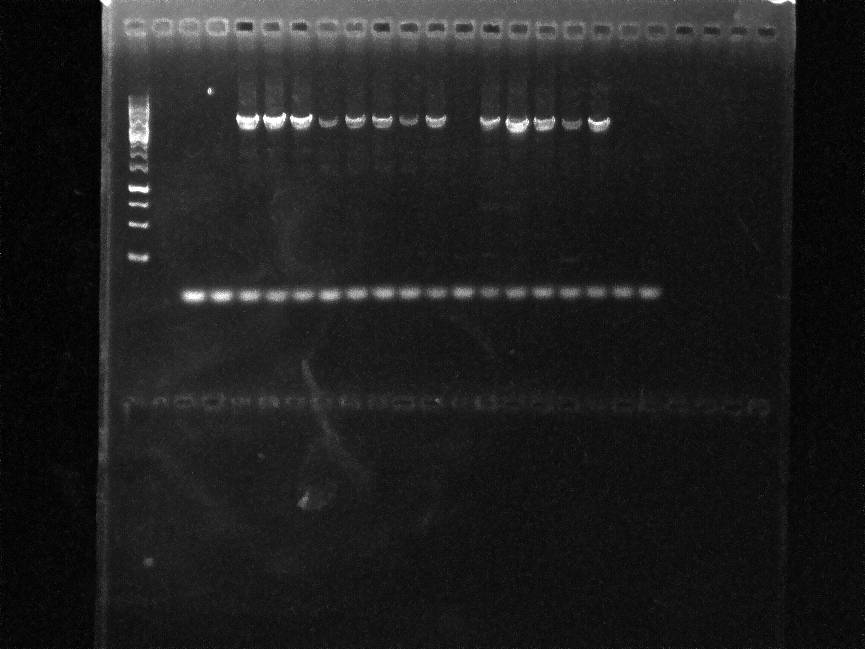

Supplement: Supplementary file 7 — Source data [file 41467_2024_46314_MOESM7_ESM.zip › Source data /Gels/Supplementary Figure 1A-gel.JPG]

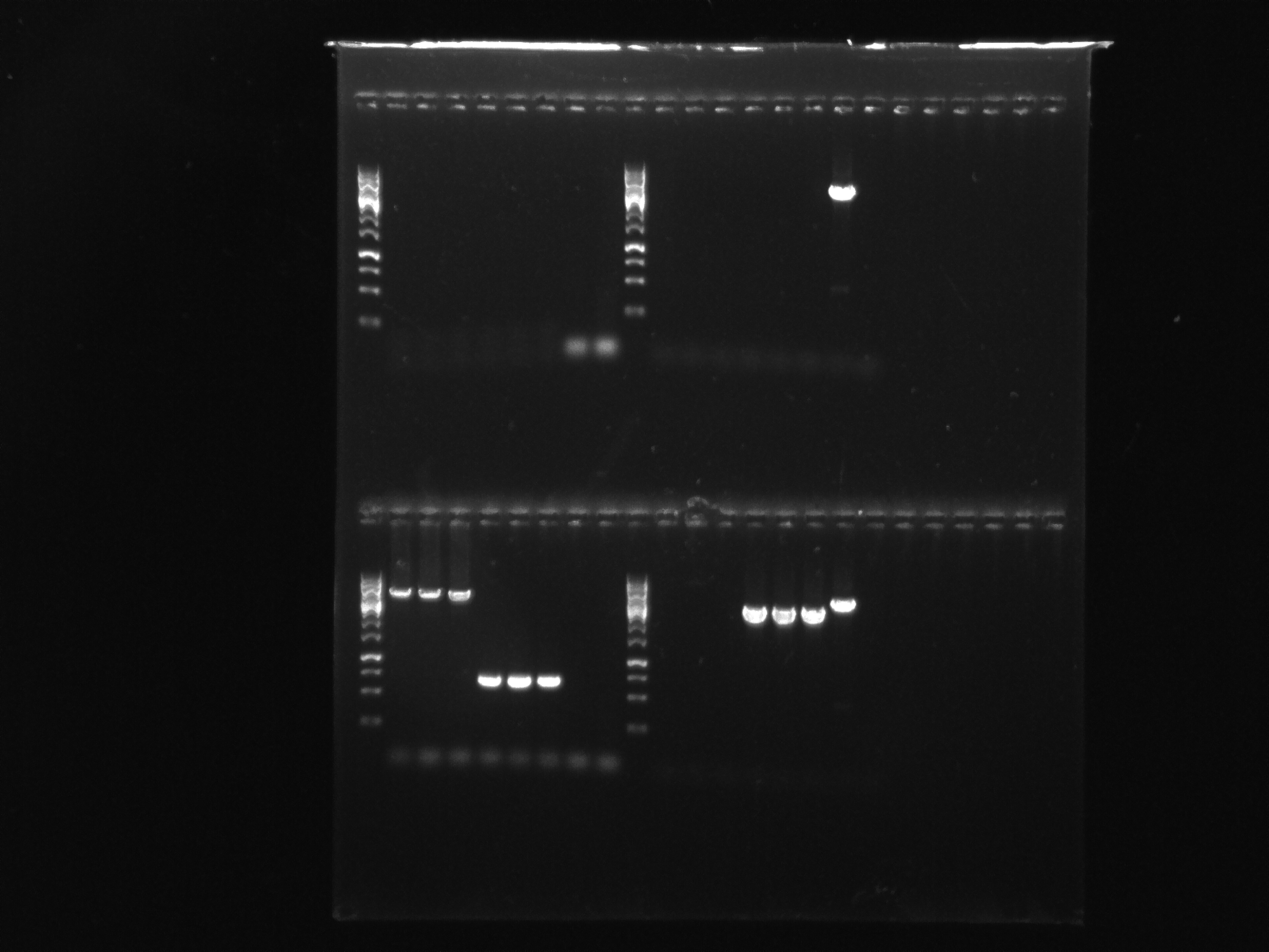

Supplement: Supplementary file 7 — Source data [file 41467_2024_46314_MOESM7_ESM.zip › Source data /Gels/Supplementary Figure 1C-gel.TIF]

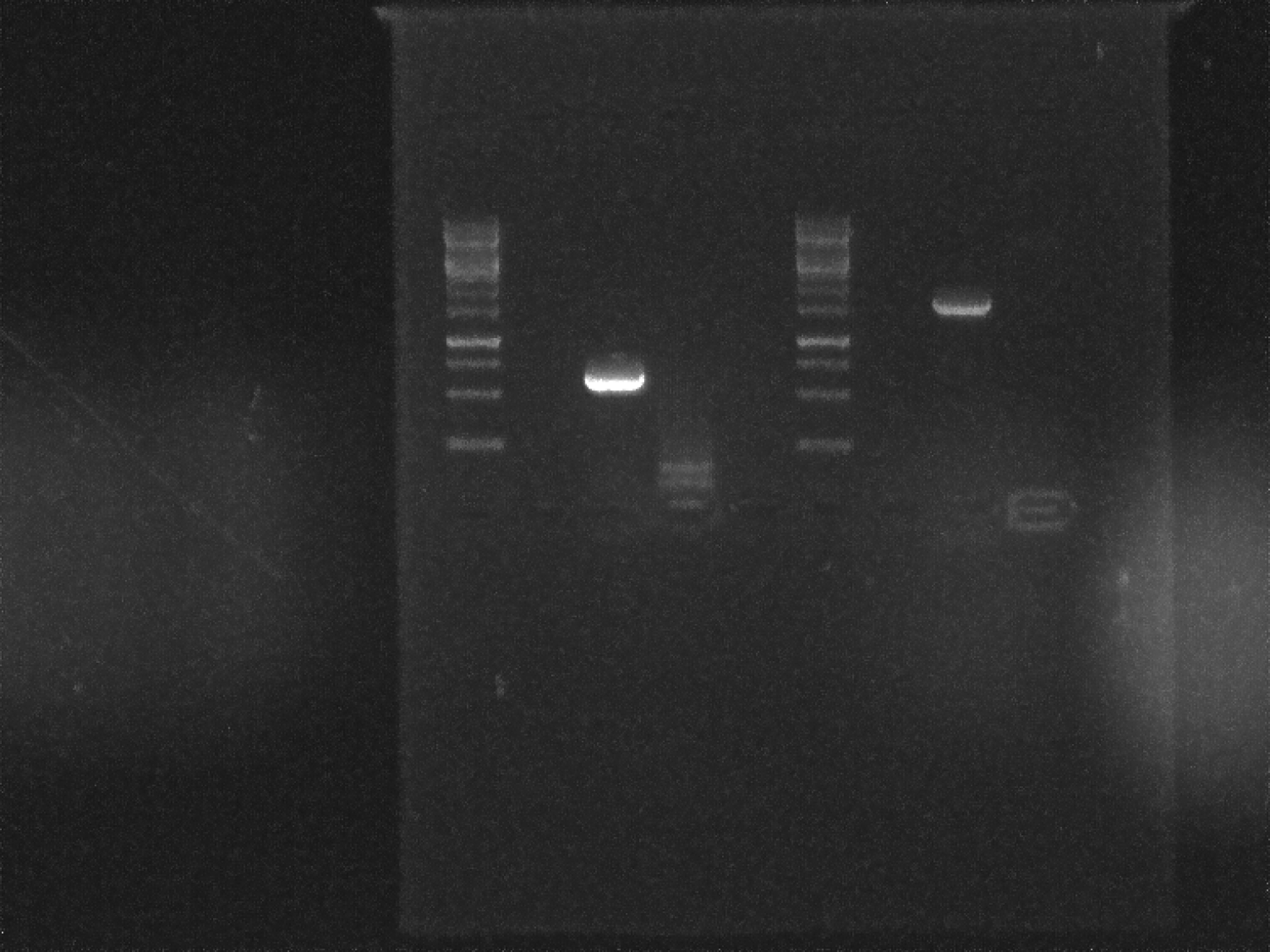

Supplement: Supplementary file 7 — Source data [file 41467_2024_46314_MOESM7_ESM.zip › Source data /Gels/Supplementary Figure 6D-gel.TIF]

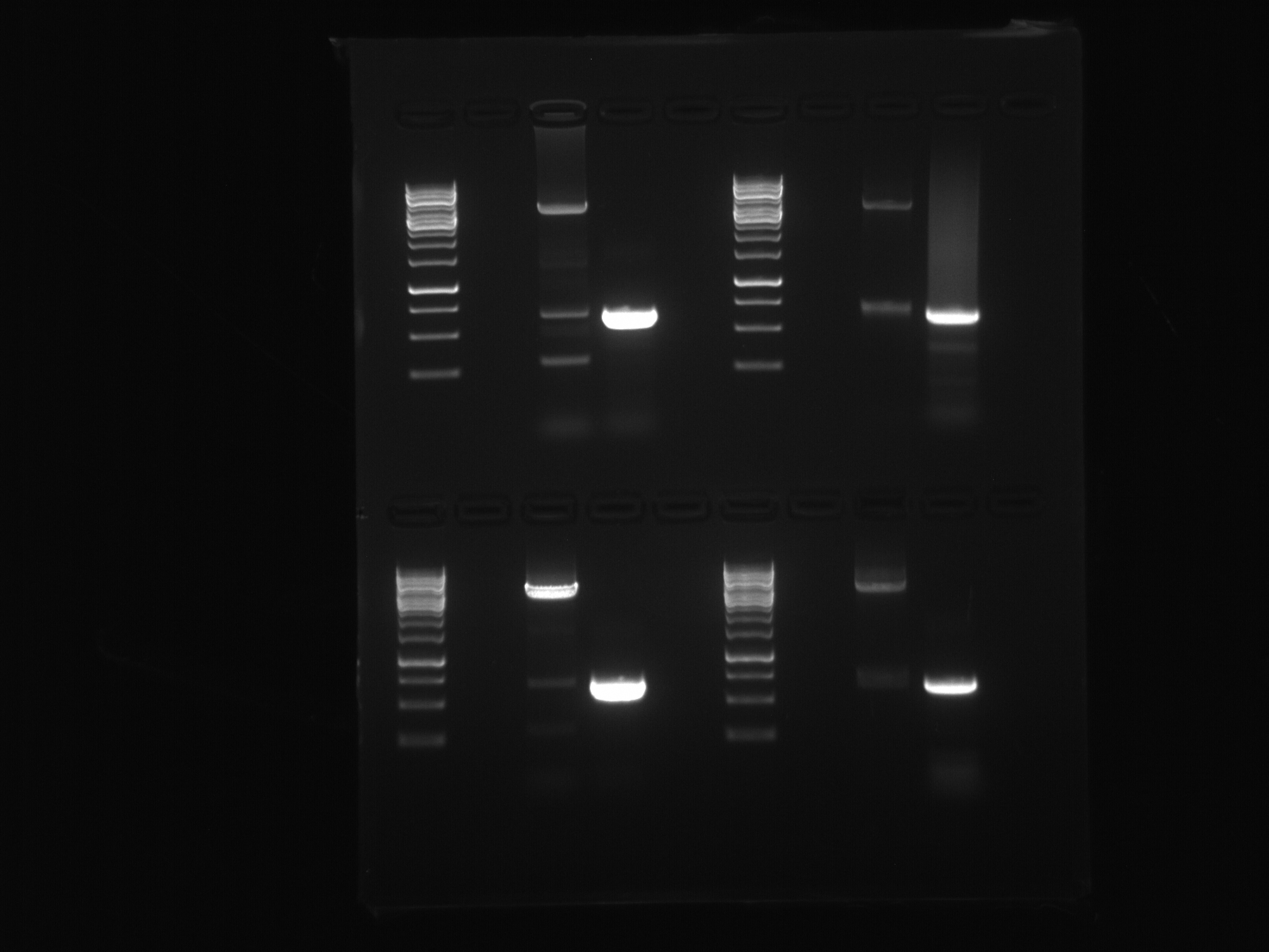

Supplement: Supplementary file 7 — Source data [file 41467_2024_46314_MOESM7_ESM.zip › Source data /Gels/Supplementary Figure 7-gel_2.TIF]

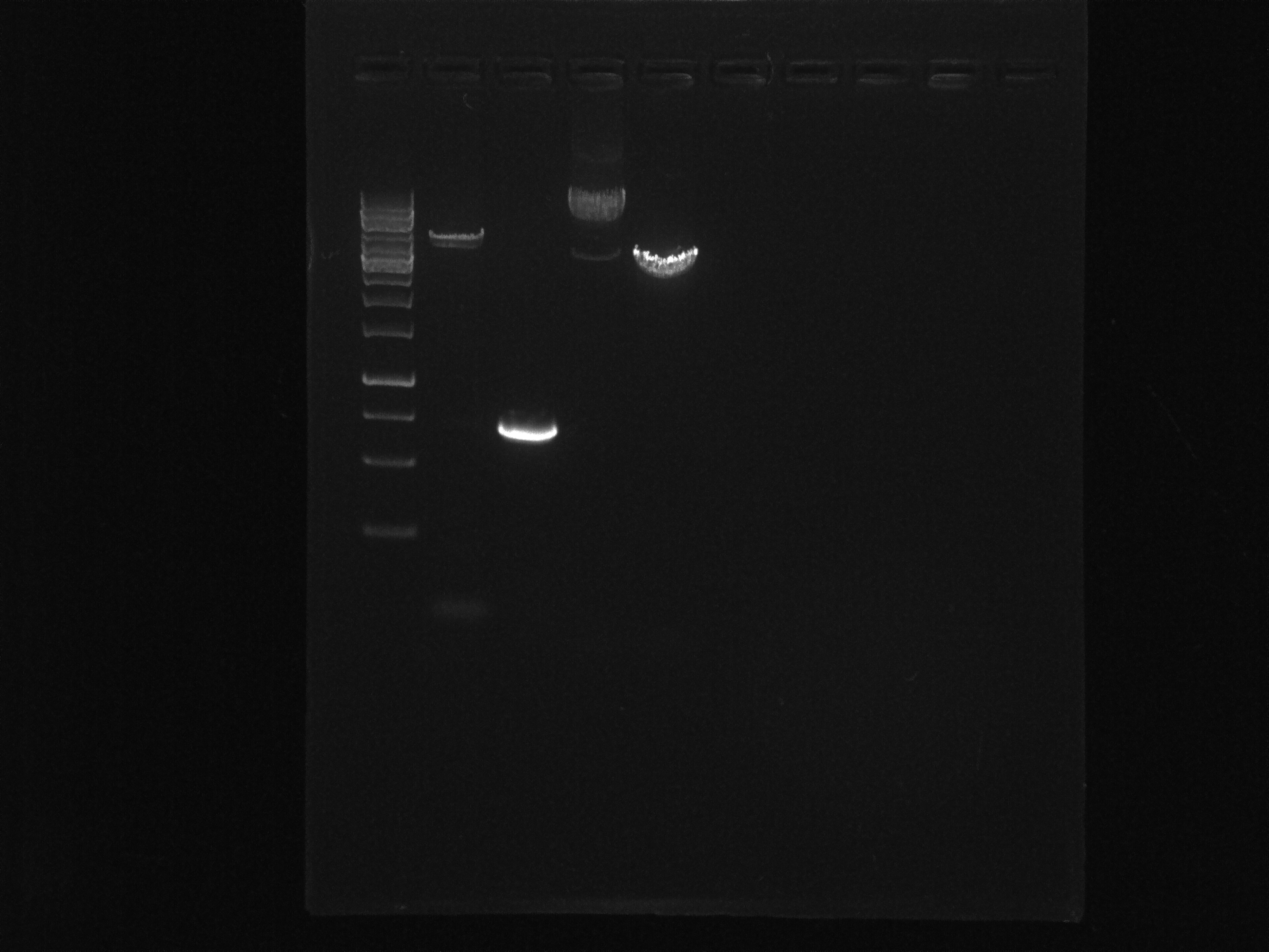

Supplement: Supplementary file 7 — Source data [file 41467_2024_46314_MOESM7_ESM.zip › Source data /Gels/Supplementary Figure 5C-gel.TIF]

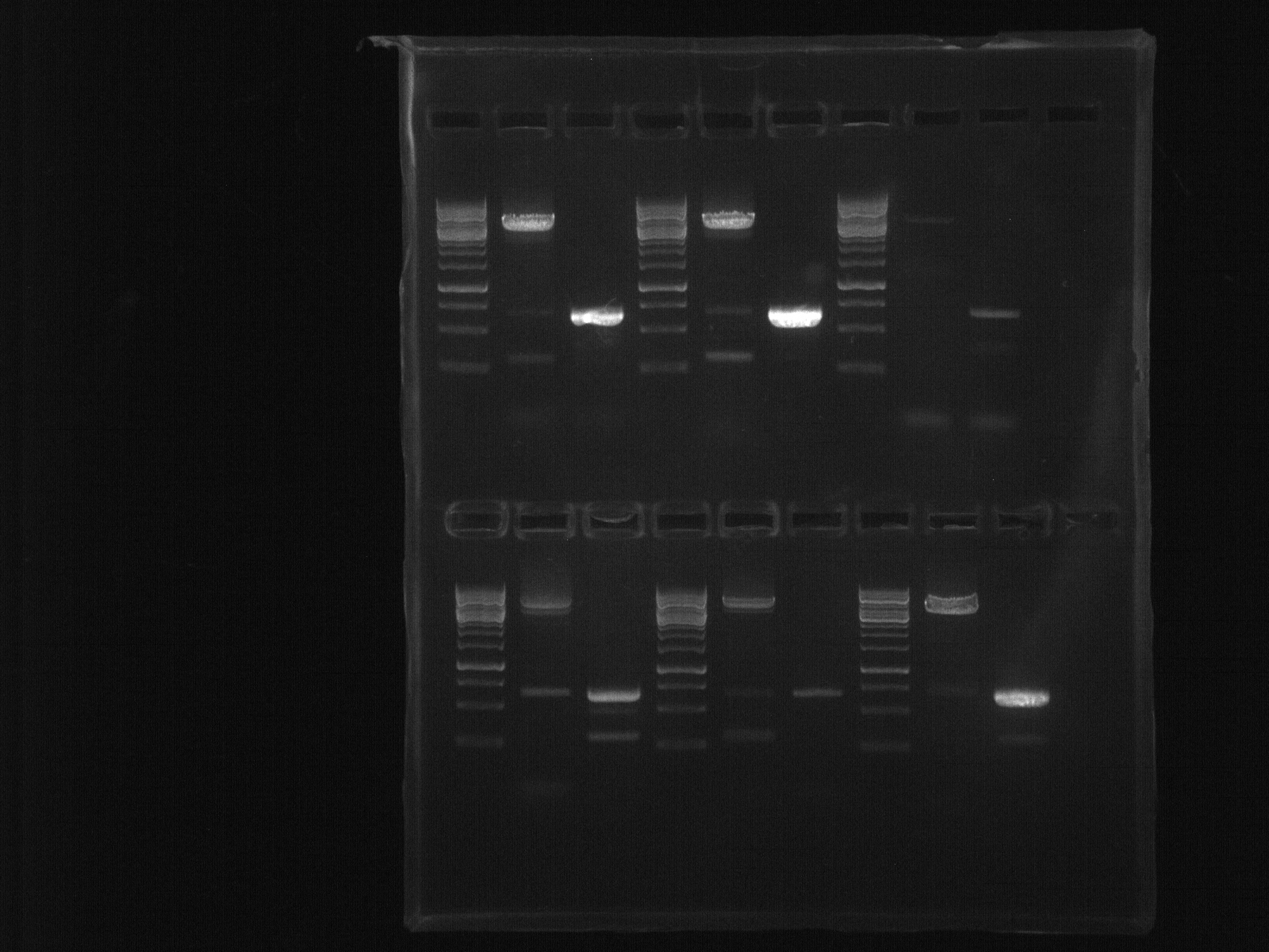

Supplement: Supplementary file 7 — Source data [file 41467_2024_46314_MOESM7_ESM.zip › Source data /Gels/Supplementary Figure 7-gel_1.TIF]

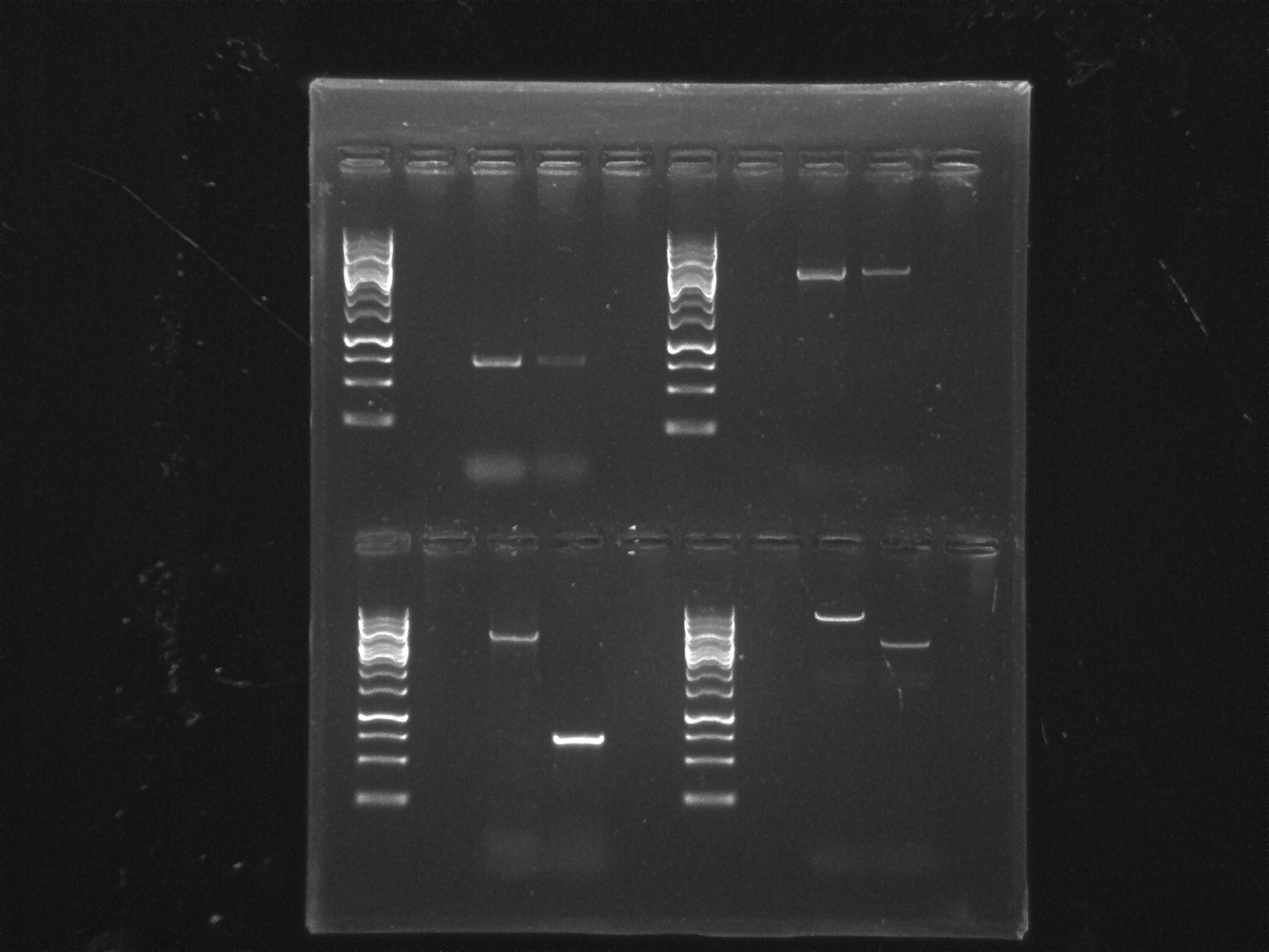

Supplement: Supplementary file 7 — Source data [file 41467_2024_46314_MOESM7_ESM.zip › Source data /Gels/Fig1J-gel.jpg]

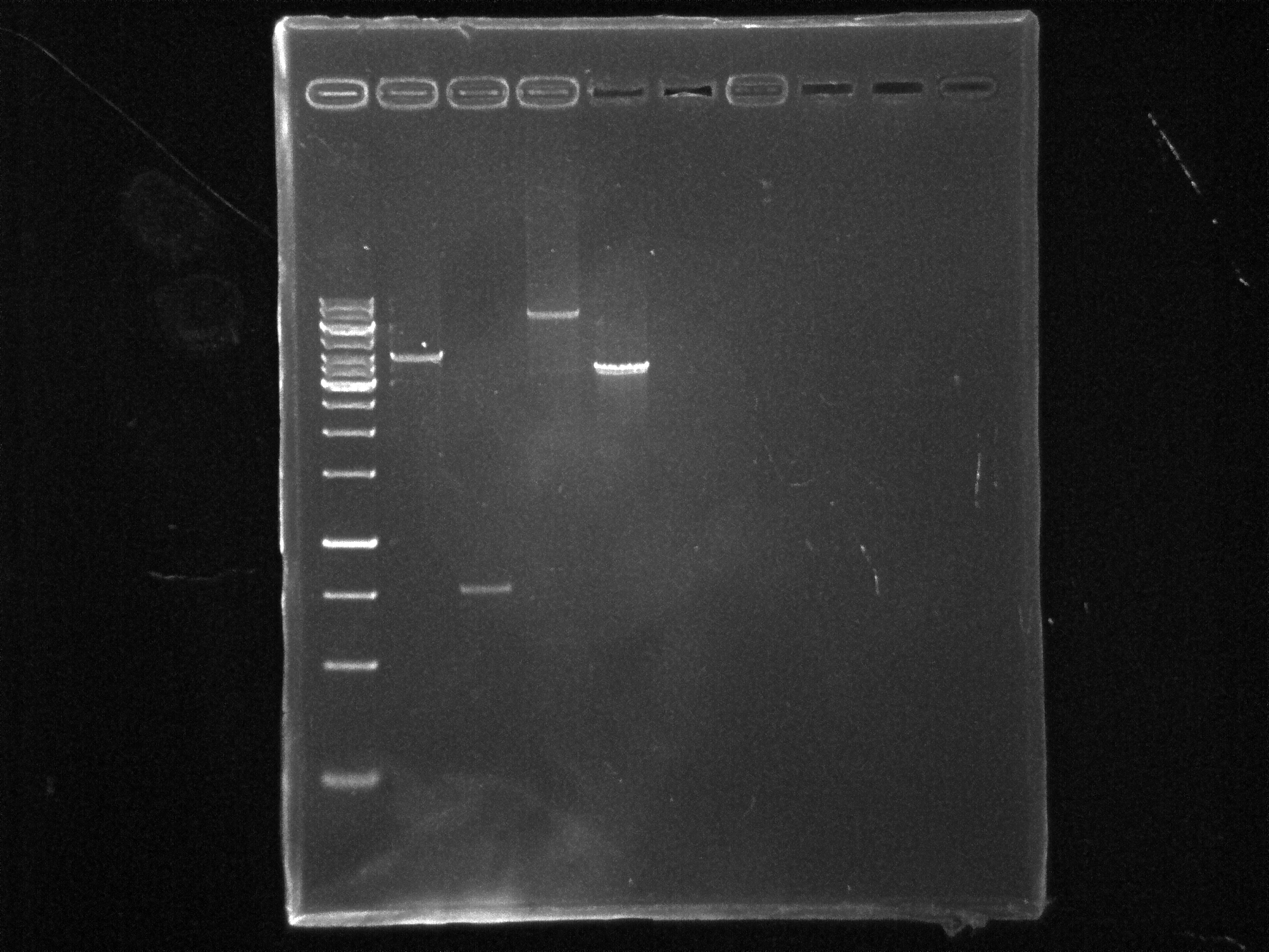

Supplement: Supplementary file 7 — Source data [file 41467_2024_46314_MOESM7_ESM.zip › Source data /Gels/Supplementary Figure 5H-gel.TIF]
